# The impactful role of the HDACs in the regulation of gene expression and as targets for disease therapy

**DOI:** 10.1126/sciadv.aee7295

**Published:** 2026-06-05

**Authors:** Bryce A. Van Bree, Lillian J. Eichner

**Affiliations:** Department of Biochemistry and Molecular Genetics, Northwestern University, 303 E Superior Street, Chicago, IL, USA.

## Abstract

Histone deacetylases (HDACs) are potent regulators of gene expression, yet incomplete understanding of individual HDAC function coupled with the lack of selective inhibitors has impeded successful targeting of these proteins for the improvement of human health. Although all HDAC proteins contain a deacetylase domain, the 18 distinct human HDAC proteins have remarkably distinct mechanisms of action. In a misleading oversimplification, HDACs are often thought to uniformly deacetylate histones to affect global gene expression in a functionally redundant manner. Instead, findings continue to define the distinct roles of individual HDACs, highlighting the potential value of data-driven precision targeting of individual HDACs or specific subclasses of HDACs for disease therapy. Here, we will discuss this decade’s advances about how the zinc-dependent HDACs, HDAC1 to HDAC11, regulate gene expression in health and disease.

## Teaser

Renaissance of HDACs as impactful regulators of gene expression, which positions them as promising targets for disease therapy.

## INTRODUCTION

Histone deacetylases (HDACs) are dominant regulators of gene expression. A major mechanism of gene expression control imparted by HDACs is posttranslational modifications (PTMs) of amino acid residues in histone tails. Histone PTMs can tighten and relax chromatin to change the accessibility of gene loci to transcriptional machinery, thereby regulating levels of gene expression (*1*–*3*). Canonically, HDACs remove acetyl groups from histones to alter chromatin structure and regulate gene expression. The positive charge associated with deacetylated lysines results in enhanced affinity between histones and negatively charged DNA, resulting in restricted chromatin accessibility and down-regulated gene expression (*4*–*7*). However, despite their classical characterization as histone lysine deacetylases, the HDACs also deacetylate key lysine residues of nonhistone proteins in molecular mechanisms essential to physiology and disease (*4*, *8*). In addition, some HDACs also function via molecular mechanisms independent of their enzymatic activity (*4*, *9*, *10*).

In humans, there are 18 distinct HDAC proteins, or lysine deacetylases (KDACs), that have myriad epigenetic regulatory functions in many distinct contexts of physiology and disease (*4*–*6*, *9*). HDACs are further divided based on their required cofactor binding and the evolutionary conservation of their catalytic deacetylase domains. HDAC1 to HDAC11, which encompass class I, II, and IV HDACs, are highly evolutionarily conserved and require zinc for their catalytic activity and/or structural stability (*7*, *11*–*17*). The other seven HDACs require nicotinamide adenine dinucleotide (NAD) and are evolutionarily distinct from the zinc-dependent HDACs (*18*, *19*). The NAD-dependent HDACs, known as the class III HDACs or sirtuins (SIRT1 to SIRT7), have important roles in physiology and disease reviewed elsewhere (*18*, *19*), but will not be discussed further here.

**Fig. 1.**
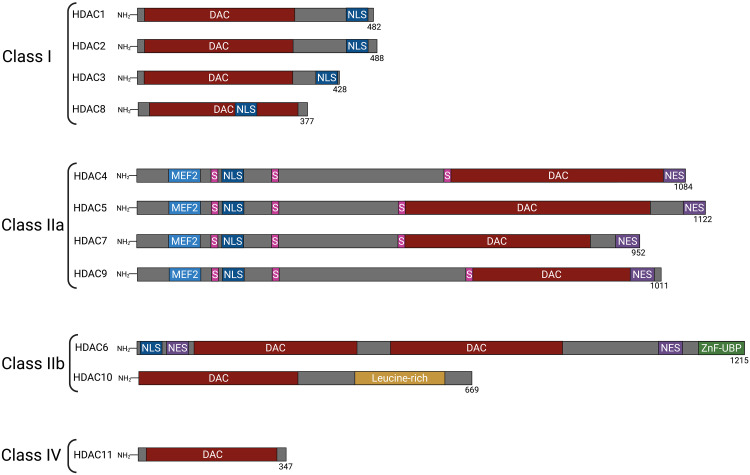
The zinc-dependent HDAC superfamily. DAC, deacetylase domain; NLS, nuclear localization sequence; NES, nuclear export sequence; S, phosphorylated serines associated with 14-3-3 binding; MEF2, MEF2-binding domain; ZnF-UBP, zinc finger ubiquitin binding domain; Leucine-rich, leucine-rich repeat domain. Bottom right, protein length in amino acids; the longest known isoform is represented. Created in BioRender. Van Bree, B. (2026). https://BioRender.com/d8at48z.

In this review, we will discuss recent findings about the zinc-dependent class I, II, and IV HDACs, which are further subdivided based on sequence homology to the yeast deacetylases Rpd3 and Hda1 (Fig. 1). The class I HDACs (HDAC1, HDAC2, HDAC3, and HDAC8) share homology with Rpd3, and the class II HDACs (HDAC4, HDAC5, HDAC6, HDAC7, HDAC9, and HDAC10) share homology with Hda1. Class II is further subdivided into the class IIa HDACs (HDAC4, HDAC5, HDAC7, and HDAC9) and the class IIb HDACs (HDAC6 and HDAC10) based on structural similarity of their functional domains. The class IIa HDACs feature an extended N-terminal adapter domain, while the class IIb HDACs feature an extended C-terminal domain. Structurally, HDAC6 and HDAC10 differ considerably; HDAC6 has two deacetylase domains and a zinc finger ubiquitin-binding domain at its C terminus, while HDAC10 has a single deacetylase domain and a leucine-rich repeat domain at its C terminus (*9*). The lone class IV HDAC, HDAC11, shares sequence similarity in its catalytic pocket with mammalian class I and class II HDACs (*17*).

While the potent impact of HDACs on biology is widely appreciated, several barriers stemming from incomplete understanding of multifaceted HDAC functions have impeded successful targeting of these proteins for the improvement of human health. These include (i) limiting on-target toxicity of pan-HDAC inhibitors; (ii) lack of clinically viable, isoform-specific HDAC inhibitors; and (iii) failure to elicit desired therapeutic response when used as single-agent therapeutics. Specifically, progress overcoming these barriers has been facilitated by a notable convergence between newfound depth of biological understanding, maturation of model systems, accessibility of genome-wide disease datasets such as The Cancer Genome Atlas, and development of a breadth of technologies including next-generation sequencing applications and CRISPR. The resultant enhanced understanding of HDAC function is revealing new approaches for overcoming the barriers that heretofore impeded successful targeting of these proteins and, thereby, has repositioned HDACs as promising targets in disease.

The zinc-dependent HDACs first stoked excitement more than 3 decades ago as druggable molecules whose inhibition could reprogram the epigenetic landscape correlated with cancer (*20*–*22*). Initial success of pharmacologically targeting HDACs to treat cutaneous T cell lymphoma resulted in Food and Drug Administration approval of the pan-HDAC inhibitor vorinostat in 2006. However, the therapeutic utility of HDAC inhibition (HDACi) did not meet expectations due to the limiting toxicity of pan-HDAC inhibitors coupled with disappointing single-agent therapeutic efficacy. Most HDAC inhibitors tested clinically, pan-HDAC or subclass-specific inhibitors, have not been selective for individual HDAC isoforms. Although their nomenclature suggests redundancy, the HDAC protein family is remarkably heterogeneous in structure, mechanism of action, and function. While a few specific HDAC isoforms are functionally redundant within subfamilies, most HDACs cannot compensate for loss of function of other HDACs. The barriers to successfully targeting HDACs have arisen largely due to insufficient understanding of the nonredundant, context-specific functions of individual HDAC isoforms, including functions of the multiprotein complexes in which the HDACs participate. Excitingly, distinct biological functions of individual HDAC isoforms continue to be uncovered, and more selective isoform-specific HDAC inhibitors are being developed. The papers reviewed here advance the field’s understanding of the context-specific roles of individual HDAC isoforms, and these advances are poised to help overcome barriers to successful HDAC targeting in the clinic. A new HDAC paradigm coupled with considerable advances in disease genetics and precision medicine strongly suggest that it is time to revisit the potential of targeting HDACs for the treatment of specific diseases; it is time for a new dawn.

Here, we will discuss advances since 2020 about each HDAC subfamily, the class I HDACs, class II HDACs, and class IV HDACs, as impactful regulators of gene expression in physiology and disease across a breadth of tissue contexts. The magnitude of papers published in this time frame prevent discussion of all papers on this topic; thus, a systematic framework of selection criteria was developed. We discuss primary research papers from the last 6 years that study any one of the HDACs with regard to function associated with gene expression control in physiology and disease contexts, and we prioritized discussion of papers that applied multiple orthogonal approaches centered on HDAC perturbation. Papers predicated solely on data generated using an HDAC inhibitor were excluded from discussion herein. Therefore, this review does not encompass the exhaustive body of HDAC literature but rather highlights emerging themes with attention to findings that implicate individual HDACs as promising targets for disease therapy.

## RESULTS AND DISCUSSION

### Class I HDACs: HDAC1, HDAC2, HDAC3, and HDAC8

**Fig. 2.**
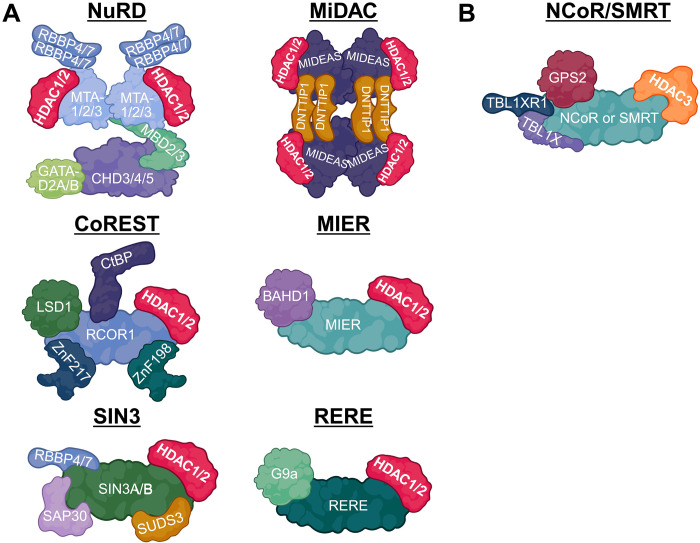
The multiprotein complexes containing HDAC1/HDAC2 and HDAC3. (**A**) HDAC1/2 containing complexes. NuRD, the nucleosome remodeling and deacetylase complex; CoREST, the corepressor of RE1-silencing transcription factor complex; SIN3, the switch-independent 3 complex; MiDAC, the mitotic deacetylase complex; MIER, the mesoderm induction early response complex; RERE, the arginine–glutamic acid dipeptide repeats complex. (**B**) HDAC3 containing complex. NCoR/SMRT, the nuclear receptor corepressor and silencing mediator of retinoic acid and thyroid hormone receptor complex. Created in BioRender. Van Bree, B. (2026). https://BioRender.com/b2v1b7s.

**Fig. 3.**
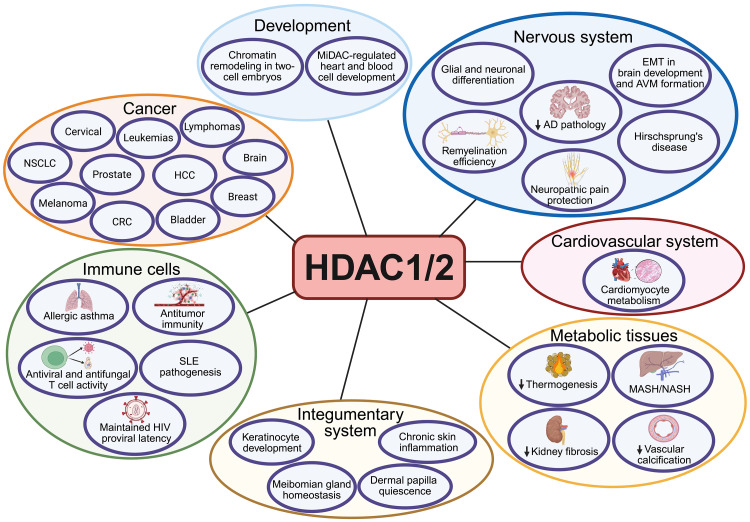
Findings define previously unidentified roles for HDAC1 and HDAC2 in physiology and disease. Arrows indicate directionality associated with HDAC1/HDAC2 activity. Created in BioRender. Van Bree, B. (2026). https://BioRender.com/l6hw3f9.

**Fig. 4.**
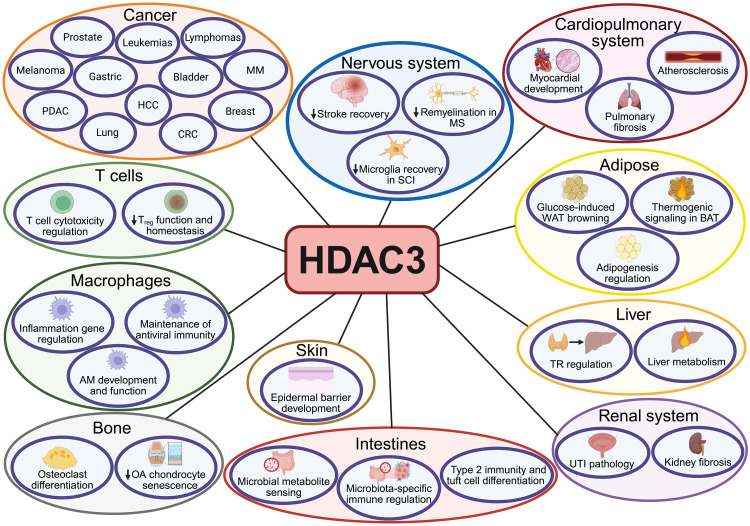
Findings define previously unidentified roles for HDAC3 in physiology and disease. Arrows indicate directionality associated with HDAC3 activity. Created in BioRender. Van Bree, B. (2026). https://BioRender.com/umh6pvq.

**Fig. 5.**
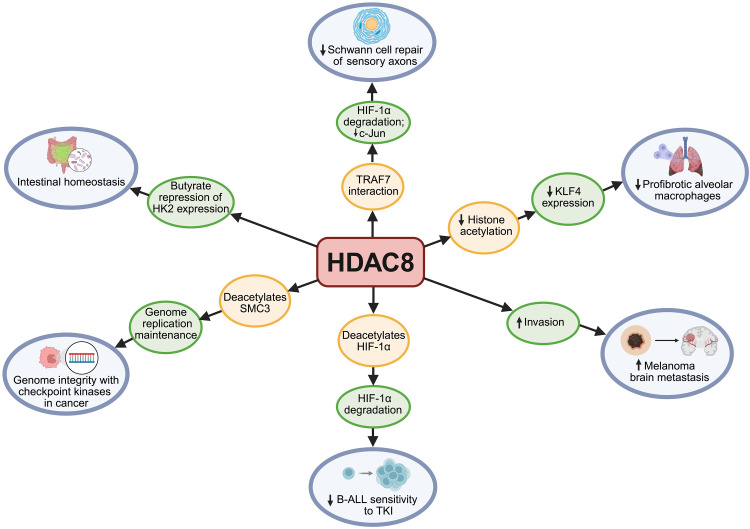
Findings define previously unidentified roles for HDAC8 in physiology and disease. Arrows indicate directionality associated with HDAC8 activity. Created in BioRender. Van Bree, B. (2026). https://BioRender.com/u90y8pa.

The class I HDAC subfamily comprises HDAC1, HDAC2, HDAC3, and HDAC8. Class I HDACs are defined by their homology to the yeast HDAC Rpd3 along with their highly conserved deacetylase domains and primarily localize to the nucleus (*4*, *7*, *9*, *11*, *12*, *16*). HDAC1, HDAC2, and HDAC3 function as key components of multiprotein complexes (*9*, *23*). HDAC1 and HDAC2 have been found to participate in multiple multiprotein chromatin modifying complexes including the corepressor of RE1-silencing transcription factor (CoREST), nucleosome remodeling and deacetylase (NuRD), switch-independent 3 (SIN3), mitotic deacetylase (MiDAC), mesoderm induction early response (MIER), and arginine–glutamic acid dipeptide repeats (RERE) complexes (Fig. 2). In contrast, HDAC3 is the primary endogenous HDAC associated with the nuclear receptor corepressor and silencing mediator of retinoic acid and thyroid hormone receptor complex (NCoR/SMRT) complex, and cooperation with the NCoR/SMRT complex is required for HDAC3 catalytic activity (Fig. 2) (*9*, *23*). Studies also continue to identify interactions between HDACs and new proteins in incompletely characterized complexes with gene regulatory capabilities, indicating opportunity for continued discovery. Whereas HDAC1 and HDAC2 impart function primarily by deacetylating histones, established HDAC3 mechanisms of action include deacetylating histones, nonhistone proteins, and noncatalytic mechanisms (*9*, *23*). HDAC1 and HDAC2 largely do not share functional redundancy with HDAC3. Here, we discuss findings on class I HDAC regulation of gene expression across many contexts of physiology and disease (Figs. 3 to 5). These findings advance our understanding of the context-specific roles of the class I HDACs and their complexes. Some drugs that predominantly inhibit class I HDAC isoforms are approved for clinical use such as romidepsin, tucidinostat, and givinostat. However, recent studies suggest that greater pharmacological selectivity for specific HDAC isoforms within class I may potentiate enhanced therapeutic benefit while minimizing unnecessary on-target toxicity associated with inhibition of multiple HDAC isoforms.

### HDAC1 and HDAC2

Findings continue to uncover roles for HDAC1 and HDAC2 in the regulation of gene expression (Fig. 3). Here, we discuss emerging areas of focus including development, nervous system, cardiovascular system, metabolic tissues, integumentary system, and the immune system. Key nonredundant roles for HDAC1 and HDAC2 in cancer continue to be defined, with particular attention to blood cancer, brain cancer, breast cancer, and colorectal cancer (CRC). New studies have also explored HDAC1 and HDAC2 mechanisms of action beyond canonical histone deacetylation.

#### Development

Findings implicate HDAC1/HDAC2 as essential regulators of gene expression during early and late embryonic development. In early embryo development, ZSCAN4, which exhibits bursts of expression in two-cell embryos during zygotic genome activation, was found to regulate chromatin remodeling (*24*). ZSCAN4 interacted with corepressors HDAC1, LSD1, and tripartite motif-containing protein 28 (TRIM28) to transiently form a repressive chromatin complex and then recruits the E3 ubiquitin ligase TRIM25 to mediate the degradation of these chromatin repressors (*24*). In a separate study, sodium butyrate treatment was found to increase the population of 2C-like cells and enable reprogramming of embryonic stem cells into trophoblast stem cells by inhibiting the HDAC1/2-LSD1 complex (*25*). A study of the MiDAC complex identified that mice lacking MiDAC proteins die during late embryogenesis due to perturbations in gene expression that result in heart malformation and hematopoietic failure (*26*). This suggests that MiDAC has an essential and unique function that cannot be compensated by other HDAC complexes, and cryo–electron microscopy structure revealed a unique mode of assembly for MiDAC in which four copies of HDAC1 are positioned at the periphery with outward-facing active sites, suggesting that the complex may target multiple nucleosomes (*26*). Considering their essential roles in embryonic development, it follows that HDAC1 and HDAC2 are key effectors of gene expression control in multiple tissue contexts, including differentiation programs of the nervous system.

#### Nervous system

HDAC1- and HDAC2-regulated gene expression programs have been implicated in studies of the nervous system and its pathologies. In healthy neuronal function, proper axonal myelination and function of the vertebrate central nervous system rely largely on the timely differentiation of oligodendrocytes (OLs). A study identified neural zinc finger (NZF2) as a crucial orchestrator that controls the timing of OL differentiation during both development and myelin repair (*27*). NZF2 binding in the NK2 homeobox 2 (*NKX2.2*) gene locus was linked with HDAC1 disruption to coordinate OL gene expression programs (*27*). In addition, a study on cross-talk between epigenetic and splicing changes in epithelial-to-mesenchymal transition (EMT) found that the C2H2 zinc finger protein 827 (ZNF827) is induced in EMT including during brain development (*28*). ZNF827 was found to orchestrate a large-scale remodeling of the splicing landscape by recruiting HDAC1 for epigenetic modulation of distinct genomic loci, thereby slowing RNA polymerase II progression and altering the splicing of genes encoding key EMT regulators in cis (*28*). Brain cortex development was also found to have an HDAC1/2 dependency in the regulation of proper neuronal production and the integrity of cortical architecture (*29*). HDAC1 and HDAC2 double conditional knockout mice were used to establish a role for HDAC1/2 in the Wnt-dependent transition from neuroepithelial progenitor cells to radial glial progenitor cells, an essential step of neuronal generation in cortical development (*29*). Other findings identified that stimulation of neurons causes condensation of large chromatin domains, and this is accompanied by the redistribution of histone PTMs and rearrangements in the spatial organization of chromosome territories (*30*). HDAC1 was found to be a key regulator of this process, suggesting that HDAC1-dependent chromatin reorganization constitutes an important level of transcriptional regulation in neurons (*30*). Considering the broad role of HDAC1 in normal neuronal function, it follows that previously unidentified roles for HDAC1 and HDAC2 in a range of neuronal disease states have emerged.

Findings have identified roles for HDAC1 and HDAC2 in the regulation of gene expression programs involved in diseases of the nervous system including cerebral disorders, Alzheimer’s disease (AD), remyelination processes relevant to multiple sclerosis (MS), neuropathic pain, and Hirschsprung disease (HD). Cerebral cortical development in mammals involves an organized set of events including the transition of neural stem and progenitor cells from proliferative to differentiative divisions to generate neurons (*31*). A study implicated the cortex-specific PHD finger protein PHF21B in the coordination of neuronal differentiation, and *PHF21B* mutations were associated with depression and mental retardation (*31*). PHF21B was found to recruit the lysine-specific demethylase LSD1 and HDAC2 to remove monomethyl groups from histone H3 lysine 4 (H3K4) and acetylation from histone H3 lysine 27 (H3K27), respectively, to regulate cell cycle genes (*31*). A study of cerebral arteriovenous malformations (AVMs), the most common vascular malformations and the leading cause of hemorrhagic stroke, leveraged new mouse models to uncover that cerebral endothelial cells (ECs) acquired mesenchymal markers and caused vascular malformations (*32*). Changes in endothelial expression of HDAC2 altered acetyl histone H4 lysine 8 (H4K8ac) and methyl H3K27 (H3K27me), causing ECs to acquire mesenchymal characteristics and form AVMs (*32*).

Neuroinflammation occurs in many neurodegenerative diseases including AD. The transcription factor SPI1/PU.1 is located at an AD-risk genomic locus, and a screen for PU.1 inhibitors identified the A11 molecule, which was found to recruit a repressive complex containing MECP2, HDAC1, SIN3A, and DNMT3A to PU.1 motifs (*33*). Furthermore, A11 ameliorated neuroinflammation and AD pathology (*33*). DNA damage contributes to brain aging and neurodegenerative disease, and new findings identified that HDAC1 modulates 8-oxoguanine (8-oxoG) repair in the brain by supporting the activity of the DNA glycosylase OGG1 known to remove 8-oxoG associated with transcriptional repression (*34*). Correspondingly, pharmacological activation of HDAC1 was found to alleviate the deleterious effects of 8-oxoG in the 5XFAD mouse model of AD (*34*).

Many axons of the nervous system are myelinated, and without myelin, the nervous system is sensitive to degeneration (*35*). Remyelination becomes inefficient during MS, and findings implicate the acetylation state of eukaryotic elongation factor 1A1 (eEF1A1) in this process (*35*). Subcellular shuttling of acetylated eEF1A1 was found to regulate nuclear localization of SOX10, and HDAC2 contributed to deacetylation of eEF1A1 (*35*).

Peripheral nerve injury–induced neuropathic pain is a global clinical problem, and nerve injury–induced changes of gene expression in dorsal root ganglion (DRG) are critical for neuropathic pain genesis. A study uncovered that zinc finger protein 382 (ZNF382) becomes down-regulated in injured DRG neurons after nerve injury, and rescuing this attenuated nociceptive hypersensitivity (*36*). ZNF382 down-regulation was identified to disrupt the repressive complex containing HDAC1 at the silencer-promoter loop, resulting in *CXCL13* transcriptional activation, thereby implicating ZNF382 down-regulation in neuropathic pain via silencer-based regulation of CXCL13, a key neuropathic pain player in DRG neurons (*36*). Similarly, a separate study uncovered that zinc finger protein 612 (ZFP612) becomes down-regulated in injured DRG neurons after nerve injury (*37*). Reduced ZFP612 results in loss of binding by the repressive HDAC1/HDAC2/SETDB1 complex at the interleukin 1 receptor-like 1 (*IL1RL1*) gene promoter, resulting in increased *IL1RL1* expression (*37*).

HD is characterized by the absence of enteric neurons caused by the defects of enteric neural crest cells, leading to intestinal obstruction (*38*). Induced pluripotent stem cell–based models of Hirschsprung and single-cell transcriptomic analysis identified a gene set of 118 genes commonly dysregulated in all patient enteric neural crest cells and raised the implication that HDAC1 may be a key regulator of these genes (*38*). Together, these findings highlight the emerging relevance of HDAC1 and HDAC2 in nervous system disorders. It will be critical to discern whether targeting HDAC1 and HDAC2 for treatment of neuronal disease will prove feasible within a therapeutic window that avoids deleterious impacts on healthy tissue including in the nervous system, cardiovascular system, and metabolic tissues.

#### Cardiovascular system

Previously unidentified roles have been identified for HDAC1 and HDAC2 in cardiomyocyte development and disease. In early cardiac development, cardiomyocytes shift from anaerobic glycolysis for adenosine triphosphate (ATP) production to mitochondrial oxidative phosphorylation (OXPHOS) (*39*). In mouse embryos, HDAC1 and HDAC2 were found to promote the shift to OXPHOS by repressing cryptic transcription to maintain transcriptional fidelity of gene programs necessary for mitochondrial biogenesis (*39*). HDAC1 and HDAC2 were identified as regulators of mammalian cardiac bioenergetic maturity through their repression of these cryptic transcripts in developing cardiomyocytes (*39*). During heart failure, cardiomyocytes metabolically shift away from fatty acid oxidation (FAO) toward glycolysis for ATP production (*40*). In heart failure, HDAC1 was found to be recruited by the transcriptional repressor HEY2 (Hairy/enhancer-of-split related with YRPW motif 2) to regulate expression of metabolic genes implicated in cardiac energy homeostasis (*40*). These genes were transcriptionally repressed by HEY2 upon cardiac stress to reduce mitochondrial FAO and induce glycolysis, raising implications for the HEY2-HDAC1 axis in heart failure (*40*). HDAC1 and HDAC2 have emerged as key regulators of metabolic gene expression in the cardiac context, roles paralleled in other metabolic tissues as well.

#### Metabolic tissues

HDAC1 and HDAC2 have been found to have key epigenetic roles in adipose tissue function, liver pathology, and chronic kidney disease (CKD). Unlike white adipocytes, brown and beige adipocytes are capable of thermogenesis, and this thermogenic capacity may harbor therapeutic potential in a variety of metabolic disorders (*41*). In beige adipocytes, HDAC1 was found to cooperate with the transcription factor Ten-eleven translocation 1 (TET1) to suppress expression of the thermogenic genes uncoupling protein 1 (*UCP1*) and peroxisome proliferator-activated receptor gamma (PPARγ) coactivator 1-alpha (*PPARGC1A*) to calibrate thermogenesis (*41*).

In liver, HDAC1 was found to contribute to metabolic dysfunction–associated steatohepatitis (MASH) pathology by deacetylating the gluconeogenesis-regulating transcription factor Forkhead box O1 (FOXO1) and in hepatic macrophages by maintaining acetylation of FOXO1 to protect against MASH progression (*42*). MASH is an intermediate step in the progression from metabolic dysfunction–associated fatty liver disease (MASLD) to cirrhosis, and roles for HDAC1 and HDAC2 were identified (*43*). HDAC1 and HDAC2 were recruited to the promoters of MASH-associated genes *CD36* and *CCL2* by methyltransferase like 3 (METTL3), an RNA methyltransferase that catalyzes mRNA m^6^A modifications, to deacetylate H3K9 and H3K27 and suppress the expression of *CD36* and *CCL2* (*43*). METTL3 was identified as a negative regulator of NASH pathogenesis through protective HDAC1- and HDAC2-mediated suppression of fatty acid uptake and inflammation-inducing gene expression (*43*). Another study corroborated the role of HDAC1 in suppressing the expression of *CD36* and *CCL2* to protect against MASH progression in conditional knockout mouse models for Wilms’ tumor 1–associating protein (WTAP), a nuclear protein and regulator of mRNA m^6^A modification and splicing (*44*). In primary hepatocytes, nuclear WTAP was found to be in complex with METTL3 and HDAC1, suggesting a mechanism of MASH progression whereby HDAC1 fails to be recruited by WTAP and METTL3, thereby allowing expression of genes associated with white adipose lipolysis, hepatic free fatty acid uptake, and inflammation characteristic of MASH (*44*). A separate study found that HDAC1 associated with upstream stimulatory factor 2 (USF2) to inhibit expression of lysosomal and autophagy genes under nutrient-rich conditions (*45*). HDAC1 and USF2 bound to the CLEAR motif in lysosomal and autophagy genes reduced H3K27 acetylation and down-regulated gene expression (*45*). Although experiments were predominantly carried out in mouse embryonic fibroblasts, findings were validated in HepG2 liver cancer cells (*45*).

In vascular smooth muscle cells (VSMCs), osteogenic transdifferentiation is the primary mechanism underlying vascular calcification, a complication associated with CKD and diabetes mellitus (*46*). In human primary VSMCs, HDAC1 and HDAC2 associated with DNA demethylation enzyme TET2, a transcriptional repressor of Runt-related transcription factor 2 (*RUNX2*), which is a necessary and sufficient regulator of VSMC osteogenic differentiation (*46*). In CKD, a study identified endogenous regulators of transforming growth factor–β (TGF-β), which drives fibrosis, and implicated KLF13 as a regulator of tubulointerstitial fibrosis (*47*). KLF13 recruited the HDAC1-containing SIN3A repressor complex to limit expression of the profibrotic genes induced by TGF-β (*47*). Together, findings highlight HDAC1 and HDAC2 as potent regulators of metabolic gene expression across multiple metabolic tissues, highlighting key interactions with METTL3 and TET proteins.

#### Integumentary system

HDAC1 and HDAC2 have been implicated in skin stem cell homeostasis, Meibomian gland homeostasis, and pathways involved in chronic skin inflammation. The transcription factor ΔNp63 regulates epithelial stem cell function and maintains the integrity of stratified epithelial tissues, and findings identified a functional link between ΔNp63 transcriptional activity and expression of the long noncoding RNA NEAT1 in proliferating keratinocytes (*48*). ΔNp63 was found to repress the expression of NEAT1 by recruiting HDAC1 to the NEAT1 proximal promoter, implicating HDAC1 in the regulatory process governing epidermal morphogenesis (*48*). While cell division facilitates self-renewal and differentiation of stem cells, dormancy is required to maintain the stem cell niche (*49*). Findings identified that HDAC1 and HDAC2 maintain quiescence of the hair follicle mesenchymal niche, the dermal papilla (DP) (*49*). In the DP, HDAC1 and HDAC2 were found to suppress the expression of cell-cycle genes and promote survival of DP cells throughout the hair cycle (*49*). During the growth phase, HDAC1 and HDAC2 were found to participate in orchestrating the hair cycle clock by maintaining physiological levels of Wnt signaling in the vicinity of the DP (*49*). HDAC1/HDAC2 and HDAC3 were implicated in the development of dry eye disease (*50*). Using HDAC1, HDAC2, and HDAC3 single and double conditional knockout mouse models, HDAC1/HDAC2 were found to be functionally redundant and coessential in the proliferation and survival of adult Meibomian gland epithelial progenitor cells (*50*). HDAC3 was shown to be essential for limiting acinar progenitor cell proliferation and for permitting differentiation (*50*). Under chronic inflammatory conditions, the structural protein essential for skin barrier function filaggrin (FLG) becomes down-regulated (*51*). An AP-1 response element within the *FLG* promoter was found to be necessary for TNFα plus interferon-γ (IFN-γ)–induced down-regulation of *FLG* expression, and HDAC1 interaction with FRA1:c-JUN was implicated in this response, thereby linking HDAC1 to regulation of chronic skin inflammation (*51*). HDAC1 and HDAC2 do not only regulate inflammatory gene expression programs in skin cells but also play key roles in immune cell function directly.

#### Immune system

HDAC1 and HDAC2 were reported to regulate inflammatory gene expression in immune cell populations including subsets of T cells, dendritic cells (DCs), and B cells. In T cells, roles for HDAC1 and HDAC2 have been identified in allergic asthma, fungus-specific immune response, T cell exhaustion during viral infection, latency in HIV infection, and regulatory T cell (T_reg_) homeostasis in antitumor immunity.

Allergic asthma is orchestrated by type 2 cytokine-producing cells such as type 2 innate lymphoid cells and T helper 2 (T_H_2) cells along with epithelial-derived cytokines (*52*). A study of allergic asthma using a house dust mite challenge mouse model identified two proinflammatory subsets of lung pathogenic T_H_2 (pT_H_2) cells known to induce allergic inflammation and identified thymic stromal lymphopoietin (TSLP) and tumor necrosis factor receptor superfamily (TNFRSF) members as drivers toward pT_H_2 cell generation (*52*). Findings identified how TSLP and TNFRSF signaling shapes chromatin accessibility at type 2 cytokine gene loci by modulating HDAC1 repressive function (*52*). In a study on fungus-specific immune responses, HDAC1 was found to limit polarization of T cells toward T helper 17 (T_H_17) cells in a fungus-specific manner (*53*). HDAC1 restricted expression of the cytokine receptor GP130 and TGF-β receptor 2 to control IL-17A release, T cell polarization, and antifungal immune defense (*53*).

HDAC1 regulates multiple aspects of T cell response to viral infection. A role for HDAC1 in the differentiation of exhausted T (T_ex_) cells during chronic viral infection was also uncovered (*54*). HDAC1 was found to control the generation and maintenance of effector-like CX3CR1^+^ T_ex_ cells in a CD8^+^ T cell–intrinsic manner, and deletion of HDAC1 led to the expansion of an alternative T_ex_ subset characterized by high expression of T cell exhaustion markers and elevated viremia (*54*). HDAC1 bound to and facilitated an open chromatin state of effector-like signature gene loci in progenitor T_ex_ cells, thereby priming cell fate specification toward the CX3CR1^+^ T_ex_ subset (*54*). Infection with HIV-1 leads to the integration of the provirus into the host genome, resulting in maintained latency in a few CD4^+^ T cells, which presents a barrier to eradicating HIV-1 from infected individuals (*55*). HIV latency is associated with a lack of proviral gene expression, and a study uncovered a role for TRIM5α in the maintenance of viral latency (*55*). TRIM5α was found to suppress nuclear factor κB (NF-κB)– and SP1-driven gene expression by binding to and enhancing recruitment of HDAC1 to regulate local H3K9 deacetylation, suggesting that HDAC1 is directly involved in maintaining proviral latency (*55*).

A study on FOXP3^+^ T_regs_, which are essential for maintenance of immune homeostasis and self-tolerance, identified a role for the HDAC1- and HDAC2-containing CoREST complex, which also contains RCOR1, RCOR2, and LSD1 (*56*). Mice with T_reg_-specific deletion of *RCOR1* had reduced suppression of homeostatic proliferation, inability to maintain long-term allograft survival despite costimulation blockade, and enhanced antitumor immunity in syngeneic models (*56*).

DC progenitors adapt their transcriptional program during development to generate distinct DC subsets (*57*). HDAC1 was found to control expression, chromatin accessibility, and histone acetylation of genes encoding the transcription factors IRF4, IRF8, and SPIB required for efficient development of classical dendritic cell 2 (cDC2) subsets (*57*). Without HDAC1, DCs were found to switch immunologically, enhancing tumor surveillance through increased cDC1 maturation and IL-12 production, driving T helper 1 (T_H_1)–mediated immunity and CD8^+^ T cell recruitment (*57*).

HDAC1 and HDAC2 functions in B cells and T cells have been implicated in lupus. Deficiency of DNA demethylase family members TET2 and TET3 in B cells was found to lead to hyperactivation of B and T cells, autoantibody production, and lupus-like disease in mice (*58*). CD86, a costimulatory membrane protein, is normally down-regulated in TET2/3-competent B cells following chronic exposure of self-reactive B cells to self-antigen, as in lupus-like disease, but the absence of TET2/3 in B cells prevented HDAC1/HDAC2-mediated down-regulation of CD86 expression (*58*). HDAC1 also regulates T follicular helper cell (T_FH_ cell) function, which is indispensable for the formation of germinal center (GC) reactions, whereas T follicular regulatory cells inhibit T_FH_-mediated GC responses (*59*). Aberrant activation of T_FH_ cells contributes substantially to the pathogenesis of autoimmune diseases including systemic lupus erythematosus (SLE) (*59*). Findings identified that adenovirus E4 promoter-binding protein (E4BP4) inhibits T_FH_ cell differentiation by recruiting HDAC1 and the H3K27 methyltransferase enhancer of zeste homolog 2 (EZH2) to regulate *BCL6* gene expression, and this immunological mechanism was compromised in patients with SLE (*59*). Consistent with impactful roles in multiple immunological disease states, evidence continues to define important roles for HDAC1 and HDAC2 in cancer, where the immune environment can also dictate disease outcomes.

#### Cancer

New findings have expanded the understanding of the roles of HDAC1 and HDAC2 in blood cancers, brain cancers, breast cancer, and CRC and have continued to define previously unidentified roles for HDAC1 and HDAC2 in other cancer types as well.

#### Blood cancer

HDAC1- and HDAC2-mediated gene regulation has been implicated in the pathology and mechanisms of therapeutic resistance of blood cancers including myeloid leukemias [acute myeloid leukemia (AML) and acute promyelocytic leukemia (APL)], lymphoid leukemias [B cell acute lymphoblastic leukemia (B-ALL), T cell acute lymphoblastic leukemia (T-ALL), and pediatric acute lymphoblastic leukemia (ALL)], and lymphoma [anaplastic large cell lymphoma (ALCL)].

In AML, tumor cells often exhibit increased expression of the endoplasmic reticulum (ER)–associated E3 ubiquitin ligase RING finger protein 5 (RNF5), which is implicated in ER stress response to chemotherapies (*60*). RNF5 knockout in AML cell lines was found to induce transcriptional changes that paralleled the effect of HDAC1 inhibition, and RNF5 pharmacological inhibition sensitized AML cells to HDACi (*60*). Separately, a study on the Forkhead family transcription factor FOXC1 in AML pathology identified transcriptional mechanisms governed by FOXC1 (*61*). FOXC1 is highly expressed in ∼20% of AML cases and is a key regulator of mesenchymal and mesodermal differentiation (*61*). FOXC1 was found to recruit a repressor complex containing the RUNX1 transcription factor, Groucho corepressor family member TLE3, and HDAC1 to the enhancers of genes responsible for monocyte/macrophage lineage differentiation (*61*). Because induction of differentiation is a major goal of AML treatment, findings implicate the HDAC1/RUNX1/TLE3 repressor complex as a target for eliciting desired treatment outcomes (*61*). Typical APL is characterized by the t(15;17) chromosome translocation that creates the promyelocytic leukemia and retinoic acid receptor-α fusion (PML::RARα) that dysregulates gene expression (*62*). While PML::RARα has been shown to restrict differentiation programs by repressing gene expression, a study described a mechanism where PML::RARα activated gene expression by coordinating HDAC1 and p300 at superenhancers associated with APL maintenance (*62*).

Previously unidentified roles for HDAC1 and HDAC2 in lymphoid leukemias have also been uncovered. In B-ALL, the clinical success of chimeric antigen receptor T (CAR-T) cell therapy is limited by T cell exhaustion and dysfunction, and targeting epigenetics has shown promise for overcoming CAR-T cell exhaustion (*63*). A study on class I HDACi identified that HDAC inhibitors M344 and chidamide enhanced antitumor efficacy in an HDAC1-dependent manner (*63*). CAR-T memory was maintained and exhaustion reduced via elevated expression of Wnt pathway genes. Preclinical CAR-T cell therapy together with class I HDACi significantly increased survival in NSG mouse models of B-ALL (*63*). High-risk B-ALL frequently features deletion of the *IKAROS* gene, which is associated with resistance to chemotherapy and poor prognosis. In a study on drug resistance, IKAROS was found to prevent chemotherapy resistance by repressing *BCL2-LIKE1* (*BCL2L1*) expression in coordination with HDAC1 (*64*). HDAC1 deacetylase activity was implicated in the sensitization of B-ALL to chemotherapy (*64*). IKAROS is also a tumor suppressor in T-ALL, although its regulation of transcriptional repression in T-ALL had not been fully characterized (*65*). IKAROS was identified as a global regulator of heterochromatin landscape and gene expression in leukemia through its formation of complexes with HDAC1, which were reported to regulate enhancers (*65*). In a study on relapse in pediatric ALL, necroptosis was identified as a means to overcome apoptosis evasion (*66*). Necroptosis could be induced via receptor activating protein kinase 1 (RIPK1), and HDAC2 was identified as an antagonist of RIPK1-driven cell death (*66*). Findings suggest RIPK1 activation coupled with HDAC2 inhibition as a promising therapeutic strategy for apoptosis-evasive ALL (*66*).

ALCL commonly harbors a chromosomal translocation that results in a nucleophosmin 1 (NPM1) and anaplastic lymphoma kinase (ALK) fusion, the NPM::ALK oncoprotein (*67*). A study using in vivo genetic approaches found that T cell–specific HDAC1 or HDAC2 loss at the time of tumor initiation accelerated NPM::ALK-driven lymphomagenesis. In contrast, treatment with the class I HDAC inhibitor entinostat extended survival of mice harboring NPM::ALK PDX tumors, highlighting distinct differences between tumor-intrinsic deletion and systemic treatment effects (*67*).

#### Brain cancer

HDAC1 and HDAC2 have been reported to regulate gene expression in the pathology and mechanisms of therapeutic resistance in glioblastoma (GBM) and medulloblastoma (MB). GBM remains a great unmet clinical need, and the underlying mechanisms of GBM intratumoral cross-talk remain poorly understood (*68*). HDAC1 was identified to mediate intercellular cross-talk that provokes growth of GBM edge cells to reinitiate tumor growth after resection (*68*). HDAC1 pharmacological inhibition and short hairpin RNA silencing decreased GBM growth in vitro and in vivo following surgical resection (*68*). A study of metabolic gene programs in GBM identified that pan-HDAC inhibitors romidepsin, panobinostat, and vorinostat disrupted superenhancers regulating metabolic gene expression (*69*). Pan-HDACi suppressed levels of transcription factor cellular myelocytomatosis (c-Myc), which was attributed to the observed changes in metabolic gene expression, and these effects were rescued by HDAC1/2 interference (*69*). Another study implicated HDAC2 as a regulator of gene expression and tumor growth in GBM (*70*).

MB frequently presents with elevated Sonic Hedgehog (SHH) activity, which is associated with increased transcription factor GLI1 activity causal to disease (*71*). A study on SHH-driven MB identified that GLI1 forms a trimeric complex with HDAC1 and zinc finger transcription factor Spalt-like transcriptional factor 4 (SALL4), which supports expression of GLI1-dependent gene programs (*71*). Another subset of MB is characterized by mutations in the E3 ubiquitin ligase Kelch repeat and BTB domain containing 4 (*KBTBD4*). A study of the structure and function of KBTBD4 mutations found that the interface between HDAC1 and KBTBD4 is stabilized by the MB mutations, which inserts a bulky side chain into the HDAC1 active site pocket (*72*).

#### Breast cancer

HDAC1 and HDAC2 have been identified as important regulators of gene expression relevant in antitumor immunity and hypoxic tumor microenvironments (TMEs) in breast cancer. A study on the hypoxic TME in triple-negative breast cancer (TNBC) uncovered that hypoxia-inducible factor 1α (HIF-1α) associated with HDAC1 to repress expression of *GZMB*, *IFNG*, and *TNF* genes associated with immune effector function within tumor-associated T cells and natural killer (NK) cells (*73*). Pharmacological inhibition of HIF-1α with PX478 in combination with anti–programmed death-1 (PD-1) therapy potently reduced tumor growth and extended survival in syngeneic mouse models, and HDAC1 inhibition with Entinostat in combination with anti–PD-1 resulted in tumor regression and further survival benefit (*73*). Separately, the X-box binding protein 1 (XBP1)–HDAC2-EZH2 axis was identified to epigenetically suppress expression of ΔNp63α, which was associated with inhibiting cell migration and tumor metastasis (*74*).

#### Colorectal cancer

HDAC1 and HDAC2 are key regulators of gene expression in CRC pathology via myriad mechanisms, many of which are noncanonical. Two studies on CRC characterized mechanisms through which HDAC1 or HDAC2 directly deacetylate nuclear nonhistone proteins. In one study, the valosin-containing protein (VCP)–HDAC1-FAO pathway was shown to drive CRC progression (*75*). Canonically, VCP is a component of the ubiquitin-proteasome degradation system, but nuclear VCP was shown to coordinate with HDAC1 to suppress *CPT1A* expression to affect FAO gene expression (*75*). A study of the RB pathway identified that CDK4/6 inhibition could sensitize locally advanced rectal cancer (LARC) models to the chemotherapy oxaliplatin (*76*). HDAC1 was found to repress expression of *CHEK1*, *EXO1*, *BRCA1*, and *BARD1* DNA repair genes that are coordinately regulated by RB1 and TEAD4 (*76*). A study using patient transcriptomic data and mouse preclinical anti–PD-1 therapy-refractory mouse models reported HDAC1-dependent transcriptional repression of immunosurveillance-related genes *CXCL10* and *MCL1* (*77*)*.* MCL1 depletion conferred sensitivity to anti–PD-1 in syngeneic CRC tumor models, and combining HDAC inhibitors with anti–PD-1 overcame resistance to immune checkpoint inhibition (ICI) (*77*).

Another group of new findings from CRC systems implicate class I HDACs as regulators of crotonylation and nonhistone protein deacetylation. In a study focused on lysine crotonylation, an alternative acyl PTMs on lysine residues, HDAC1 in complex with SIN3A and GAS41 was found to enhance crotonylation of H3K27 at the p21 genomic locus, thereby repressing gene expression while maintaining an open chromatin state (*78*). Knockout of GAS41, a YEATS domain-containing protein, blunted CRC xenograft tumor growth (*78*). Separately, in a study of CRC abnormal centrosome amplification observed in CRC and the associated aberrant centriole formation, lysine acetyltransferase KAT7 was found to modulate H3K14ac at promoters of genes associated with procentriole formation dependent on competitive antagonism between the crotonylated or acetylated status of KAT7 (*79*). HDAC2 was identified to deacetylate KAT7 to allow for KAT7 crotonylation, which inhibited KAT7 histone acetyltransferase activity and reduced CRC tumor cell growth (*79*). A study of antiangiogenic drugs identified that high levels of enolase 2 (ENO2) were associated with antiangiogenic therapy resistance in CRC (*80*). ENO2 results in the production of phosphoenolpyruvate (PEP), which directly binds to inhibit HDAC1. HDAC1 was found to deacetylate β-catenin at Lys^49^, repressing the β-catenin gene expression program (*80*). By inhibiting HDAC1 activity, ENO2-derived PEP enhanced β-catenin Lys^49^ acetylation, and inhibition of ENO2 sensitized drug-resistant CRC tumors to antiangiogenic therapy (*80*). A separate study on PTM of PD-L1 found that p300 and HDAC2 were responsible for directly acetylating and deacetylating, respectively, lysine-263 in the cytosolic domain of PD-L1 to regulate its nuclear translocation (*81*). Nuclear PD-L1 was found to regulate the expression of genes associated with NF-κB signaling including *BIRC3*, *RELB*, *TRAF1*, and major histocompatibility complex (MHC) class I–associated genes *HLA-A*, *HLA-B*, and *HLA-H* (*81*). Correspondingly, pharmacological inhibition of HDAC2 extended survival in combination with anti–PD-L1 ICI in a syngeneic CRC mouse model (*81*). It will be important to discern whether HDAC1 and HDAC2 use noncanonical mechanisms selectively in CRC, or whether these mechanisms also determine HDAC-dependent biological outcomes beyond the CRC setting.

#### Other cancers

Previously unidentified roles for HDAC1 and HDAC2 have also been identified in lung cancer, melanoma, cervical cancer, prostate cancer, hepatocellular carcinoma (HCC), and bladder cancer. Among patients with Kirsten rat sarcoma virus (*KRAS*) mutant lung adenocarcinoma (LUAD), co-occurring mutation in serine/threonine kinase 11 (*STK11*/LKB1) correlates with distinctly poor response to immunotherapy compared to patients with tumors wild type for *STK11* (*82*). A CRISPR screen identified HDAC1 as a key effector of STK11-specific anti–PD-1 resistance, and a selective small molecular inhibitor of the HDAC1-containing CoREST complex, TNG260, was developed to target this dependency (*82*). TNG260 sensitized *STK11*-deficient LUAD tumors in syngeneic and autochthonous genetically engineered mouse models (GEMMs) to anti–PD-1 therapy, and modified TME was confirmed in biopsies from patients with *STK11* mutant LUAD treated with TNG260 (*82*).

As in MB described above (*71*), functional interaction between class I HDACs and SALL4 has also been identified as a vulnerability in melanoma (*83*). SALL4 was found to regulate a melanoma-specific invasion program via HDAC2-mediated epigenetic silencing of proinvasive genes including nerve growth factor receptor (*NGFR)* (*83*). Correspondingly, melanoma-specific proinvasive gene programs were up-regulated upon SALL4 knockdown, pan-HDACi with panobinostat, and class I HDACi with mocetinostat (*83*). Another study identified an HDAC1/HDAC2 dependency in adult epidermal homeostasis using homozygous epidermal codeletion of HDAC1 and HDAC2 in adult mice (*84*). HDAC1/HDAC2 regulated basal cell proliferation, apoptosis, and differentiation via a p53- and p16-dependent mechanism, suggesting the potential for the therapeutic efficacy of HDAC1/HDAC2 inhibition against epidermal basal cell carcinoma progenitors based on the mutational status of p53 and p16 (*84*).

A role for HDAC1 and HDAC2 was also identified in a study on radiotherapy in cervical cancer (*85*). The cancer/testis G antigen 12 (GAGE12) was found to mediate radiotherapy resistance through the creation of open chromatin structures to enhance DNA repair efficiency in response to irradiation (*85*). GAGE12 promoted the association of HDAC1 and HDAC2 with Actin, thereby hindering the ability of HDAC1 and HDAC2 to deacetylate H3K56 (*85*). When the GAGE-associated complex was pharmacologically inhibited in vitro, radiosensitivity was restored (*85*).

In prostate cancer where both androgen receptor (AR) and mechanistic target of rapamycin (mTOR) signaling have established roles but combined inhibition strategies have severe toxicity profiles, findings identified that AR interacts with nuclear mTOR (*86*). Upon androgen treatment, the HDAC2-containing NuRD complex was recruited with mTOR and AR to genomic loci to activate androgen-mediated gene expression, suggesting HDAC2 targeting as an alternative therapeutic approach for inhibiting AR/mTOR-driven programs (*86*).

In HCC, HDAC2 was found to regulate SmD2, a core component of the spliceosome machinery, to regulate splicing of DNA repair genes including *BRCA1* and *FANC* (*87*)*.* Findings suggest that HDAC2 deacetylates SmD2 to prevent its degradation (*87*). Targeting HDAC2 was found to enhance sensitivity to poly(ADP-ribose) polymerase (PARP) inhibitors, analogous to prior findings from SmD2 depletion with PARPi (*87*).

In bladder cancer cells, a noncanonical mechanism of gene expression activation was uncovered for HDAC1 (*88*). HDAC1 exhibited intrinsic protease activity capable of cleaving histone H3 between lysine-20 and alanine-21, and localization of HDAC1 to target gene loci with CRISPR-dCas9 was associated with an active transcriptional state and greater proliferative capacity of cancer cells (*88*).

Collectively, findings suggest promise for combination treatment approaches using HDAC1- and HDAC2-targeting inhibitors. Considering that HDAC1 and HDAC2 regulate large numbers of genes in any given context, on-target toxicity is likely to pose a challenge when drugging HDAC1 or HDAC2. If optimal therapeutic windows can be achieved for isoform- and/or complex-selective HDAC1/HDAC2 inhibitors, combination approaches are poised to elicit therapeutic benefit in many cancer types.

#### Beyond histone deacetylation

Furthermore, tissue context-agnostic studies have also identified roles for HDAC1 and HDAC2 beyond canonical histone deacetylation. One study reported that HDAC1 deacetylated a key protein involved in DNA methylation maintenance, ubiquitin-like with PHD and RING finger domains 1 (UHRF1) (*89*). Deacetylation by HDAC1 was required for chromatin association of UHRF1 during S phase, which affected inheritance of global DNA methylation (*89*). Other findings suggest that the acetyltransferase p300 can catalyze the enzymatic addition of the metabolite β-hydroxybutyrate (β-HB) to histone lysines (Kbhb), and HDAC1 and HDAC2 enzymatically remove the Kbhb PTM (*90*). Another study suggested that HDAC1 to HDAC3 catalyze the addition and removal of sorbate onto histone lysines (*91*). It remains to be determined how ubiquitous these noncanonical mechanisms are, and the extent of their impact on gene expression control imparted by HDAC1 and HDAC2.

Class I HDACs have also been shown to have potent lysine delactylase activity, and several studies suggest that the downstream gene expression changes of these delactylation events may be relevant to disease states, particularly in cancer. In platinum-based chemotherapy-resistant TNBC, HDAC2 delactylated the protein METTL3 to facilitate mRNA stability and cisplatin tolerance via up-regulated N^6^-methyladenosine modifications (*92*). In bladder cancer, HDAC2 regulated global protein lysine lactylation in a manner distinct from acetylation, and the proteins with HDAC2-regulated lactylation were predominantly involved in gene expression regulation (*93*). HDAC3 has also been identified as one of the regulatory enzymes capable of removing histone lactyl groups (*94*), and in gastric cancer models, HDAC3 was reported to delactylate the DNA repair protein nibrin (NBS1) at lysine-388. NBS1 K388 is frequently lactylated in cancer cells and predicts poor patient outcome of neoadjuvant chemotherapy (*95*). In addition, HDAC3 was implicated in a study where myocardial histone lactylation was found to associate with pathological cardiac remodeling in a METTL7B-dependent mechanism (*96*). Low METTL7B and high HDAC3 abundance was associated with delactylated histones and cardiac dysfunction, while high METTL7B and low HDAC3 abundance was associated with lactylated histones and cardiac protection (*96*). The protein lysine delactylase activity of HDACs may present an emerging direction of study in a breadth of disease states.

Collectively, histone deacetylation remains the major mechanism through which HDAC1 and HDAC2 regulate gene expression, but other HDAC-catalyzed acyl PTMs are emerging as modes of gene expression regulation in physiology and disease. However, given that the current HDAC-targeting treatment paradigm pharmacologically inhibits all HDAC enzymatic activity at the DAC catalytic pocket, HDAC inhibitors could be expected to inhibit both HDAC deacetylase and delactylase activity.

Together, studies have shed more light on the context-specific roles of HDAC1 and HDAC2 and their multiprotein complexes in regulating gene expression. Promising advances have been made in enhancing understanding of the roles of HDAC1 and HDAC2 in embryonic development; the nervous system; the cardiovascular system; metabolic tissues such as the liver, kidney, and adipose tissue; the integumentary system; immunity; and myriad cancer types. HDAC1 and HDAC2 are found within distinct multiprotein complexes (Fig. 2), and functional interaction has been identified with other proteins such as SETDB1, GLI1/SALL4, RUNX1/TLE3/FOXC1, PHF21B/LSD1, and TRIM28/LSD1 (*24*, *31*, *36*, *61*, *71*). Understanding how these mechanistic determinants can be leveraged as opportunities for enhancing selectivity of interventions targeting HDAC1 and HDAC2 is a promising area for further exploration. We anticipate that continued advancement in biochemical understanding will facilitate the development of creative approaches for selectively targeting the pathogenic functions of HDAC1 and HDAC2 that will minimize on-target toxicity.

### HDAC3

HDAC3-dependent gene expression programs continue to be defined across a breadth of important biological contexts (Fig. 4). Emerging areas of focus discussed here include the nervous system, the cardiovascular system, adipose tissue, liver, the renal system, intestinal homeostasis, skin, bone, macrophages, and T cells. HDAC3 requires cooperation with the NCoR/SMRT complex to be catalytically active (*9*, *23*), a core mechanistic handle that informs multiple studies discussed herein. HDAC3 has also been associated with impactful catalysis-independent mechanisms of gene expression control. Because HDAC3 does not have a functionally redundant homolog, nor is it a ubiquitously essential gene in adult tissue, HDAC3 provides a promising opportunity for the treatment of disease, especially cancer. Findings have precipitated key roles for HDAC3 in blood cancer, breast cancer, CRC, lung cancer, and pancreatic cancer, and its relevance in other cancer contexts continues to be defined as well.

#### Nervous system

Studies of nervous system pathologies have identified key roles for HDAC3 in injury repair programs. One study reported that an essential ketone body component β-HB positively correlated with improved outcomes in patients with stroke, and promoted functional recovery in rodents with stroke during the repair phase (*97*). β-HB was found to promote stroke recovery via inhibition of HDAC2/HDAC3. HDAC2/HDAC3 were shown to regulate gene expression of *GAT1*, a key molecular substrate for network excitability and consequent stroke recovery (*97*). Another study on stroke identified a role of HDAC3 in microglia, the major brain resident immune cell that activates within minutes after ischemic stroke and serves as the first nonneuron cell that responds to injury (*98*). Using a microglial HDAC3 knockout model, HDAC3 was found to ameliorate poststroke long-term functional and histological outcomes (*98*). HDAC3 and the transcription factor PU.1 were found to coordinate proliferation and proinflammatory gene programs (*98*). The innate immune response, including microglia and macrophages, also influences neural repair after spinal cord injury (*99*). Integration of transcriptomic approaches revealed distinct gene programs in injury-activated microglia and macrophages (*99*). Using the HDAC3-selective inhibitor RGFP966, HDAC3 was implicated as a regulator of gene programs involved in the immediate response of a key microglial subtype, suggesting that HDAC3 targeting may provide means to enhance functional recovery after spinal cord injury (*99*). HDAC3 was also implicated in a different neurological disorder, MS (*100*). MS is a demyelinating disease, where the remyelination capacity of OLs is pathologically blocked, resulting in failure to properly remyelinate axons (*100*). Findings identified that mature OLs in MS lesions were epigenetically silenced, preventing myelin production (*100*). Labeling mature OLs in vivo using a transgenic reporter line facilitated identification of a small molecular inhibitor, ESI1, that stimulated myelin production from differentiated OLs, thereby reversing MS phenotypes (*100*). ESI1 was originally reported as an HDAC3 inhibitor, linking HDAC3 to the epigenetic program dictating remyelination after demyelinating injury in MS (*100*). Collectively, these findings suggest a key role for HDAC3 in neuronal injury repair programs.

#### Cardiopulmonary system

Previously unidentified roles for HDAC3 in cardiac cell development, atherosclerosis, and pulmonary fibrosis have emerged. Leveraging single-cell RNA sequencing to explore HDAC expression during human pluripotent stem cell differentiation, findings identified a link between HDAC1 and HDAC3 and the mesendoderm gene program during exit from pluripotency (*101*). Small-molecule perturbation of HDAC1 and HDAC3 induced mesoderm while impeding endoderm and early cardiac progenitor specification (*101*). Endocardial HDAC3 was found to be required for early embryo survival and proper cardiac ventricular trabeculation (*102*). Defective myocardial growth is a primary manifestation of congenital heart disease, and cardiac ECs are central to trabeculation, the formation of myocardial meshwork extending to the cardiac chamber (*102*). Endocardial knockout of HDAC3 was associated with reduced expression of extracellular matrix (ECM) components important for trabeculation (*102*). Separately, GEMMs revealed a role for HDAC3 in atherosclerosis (*103*). HDAC3 was found to deacetylate the transcription factor specificity protein 1 (SP1), and pharmacological inhibition of HDAC3 with RGFP966 in *ApoE*^−/−^ mice effectively ameliorated atherosclerosis (*103*). HDAC3 knockout mouse models and RGFP966 treatment were also used to investigate the role of HDAC3 in a bleomycin-induced model of pulmonary fibrosis (*104*). HDAC3 was shown to be profibrotic via suppression of *Nrf2* (nuclear factor erythroid–derived 2–related factor–2) (*104*). HDAC3 knockouts and pharmacological inhibition were both shown to reduce *Nrf2* suppression and normalize fibrosis, whereas the antifibrotic effects of HDAC3 inhibition were reduced in *Nrf2*-KO mice (*104*).

#### Adipose tissue

HDAC3 is established as a key effector of adipose tissue function, yet how HDAC3 molecular networks intersect to comprehensively coordinate adipose function continues to be defined. Brown adipose tissue (BAT) is a thermogenic organ, and HDAC3 is required for BAT to maintain body temperature and uncoupling protein 1 *UCP1* expression in response to acute cold exposure (*105*). HDAC3 exists in tight association with nuclear receptor corepressors (NCoR1/NCoR2), and a study reported that genetic loss of NCoR1/2 resulted in loss of HDAC3 activity and genome binding leading to deregulation of a lipid metabolism gene program (*105*). Despite commonalities between HDAC3 and NCoR programs, loss of NCoR1/2 in BAT did not phenocopy the cold sensitivity of HDAC3 BAT knockout (*105*). Instead, *NCoR1/2* knockout BAT exhibited an inflamed phenotype attributed to *MMP9* gene expression control, which integrates ECM remodeling and inflammation (*105*). While HDAC3 deficiency in BAT impairs survival in near-freezing temperatures, short-term exposure to mild cold temperatures averted lethal hypothermia of mice lacking HDAC3 in BAT (*106*). Induction and maintained elevated levels of the transcriptional activator C/EBPβ upon short-term cold exposure was identified as an HDAC3-independent cold-adaptive means of epigenomic memory (*106*). In white adipose tissue (WAT), a study found that glucose stimulated expression of cAMP response element-binding protein (CREB)/activating transcription factor (ATF) basic leucine zipper transcription factor (CREBZF) (*107*). CREBZF acetylation at lysine-208 was found to be required for glucose-induced protein stability, and HDAC3 was identified as a regulator of CREBZF acetylation implicated in coupling glucose sensing to WAT browning (*107*). In early adipogenesis, the methyltransferase SET domain–containing 5 (SETD5) was found to form a complex with NCoR and HDAC3 to prevent histone acetylation of enhancers for two master adipogenic regulatory genes *CEBPA* and *PPARG* (*108*). SETD5 protein levels were dynamically regulated, which served as a molecular switch to facilitate adipogenesis by providing spatiotemporal control of the NCoR-HDAC3 complex (*108*). Potentiated by established in vivo HDAC3 phenotypes, the adipose context has emerged as a powerful context for ongoing exploration and delineation of HDAC3-intersecting molecular networks. Mechanistic findings from adipose tissue complement substantial depth of knowledge about HDAC3 function from the liver context.

#### Liver

HDAC3 function has been well established in liver and, consequently, the liver has proven to be a valuable context for ongoing discoveries about the HDAC3 molecular network, including interplay with nuclear receptors. HDAC3 loss in liver is established to cause hepatosteatosis due to derepression of lipid metabolism genes. A study found that NCoR1 and NCoR2 double knockout in hepatocytes phenocopied the HDAC3 knockout fatty liver phenotype (*109*). In addition, NCoR1/2 knockout affected glycogen storage associated with fasting hypoglycemia, which was associated not with HDAC3 but rather loss of chromatin access affecting glucocorticoid receptor DNA binding at gluconeogenic genes (*109*). Thyroid hormones are powerful regulators of metabolism that act via the nuclear receptor thyroid hormone receptor (TR) to regulate gene expression programs (*110*). To better understand thyroid hormone gene expression control, a mouse line was generated in which endogenous TR β1 was epitope tagged to facilitate chromatin immunoprecipitation (ChIP) at endogenous, low physiological levels (*110*). In liver, TR binding was enriched at canonical motifs irrespective of the transcriptional direction of the enhancer; instead, transcriptional activity correlated with the ratio of coactivator CBP to repressor NCoR1/HDAC3, rather than their presence or absence (*110*). Separate from its interplay with nuclear receptors, loss of hepatic HDAC3 led to hepatic iron overload, ferroptosis, and liver damage via a downstream effector in the Hippo pathway, yes-associated protein (YAP) (*111*). Knockdown of both HDAC3 and YAP ameliorated iron-induced liver damage, implicating HDAC3 and the Hippo pathway in iron homeostasis and as targets for iron overload/ferroptosis-associated diseases (*111*).

#### Renal system

HDAC3 continues to be relevant in new disease contexts of the renal system. A study seeking new approaches for treatment of urinary tract infection found that HDAC3 inhibited the expression of genes that encode for ribonucleases 4 and 7 (RNase 4 and 7), both of which have antimicrobial activity against antibiotic-resistant uropathogens in murine urinary tract infections. Class I HDACi with entinostat was found to protect mice from transurethral *Escherichia coli* challenge through up-regulation of RNase 4 and 7 (*112*). While pan-HDAC inhibitors have demonstrated a role for nonspecific HDAC activity in inflammation and fibrosis, HDAC3 specifically was identified to promote renal inflammation and fibrosis through regulation of proinflammatory and profibrotic gene expression, respectively (*113*). In addition to the HDAC3-specific inhibitor RGFP966, HDAC3 conditional knockout in two mouse models of renal fibrosis was used to determine that HDAC3 contributes to renal fibrosis and inflammation (*113*). This study corroborated a prior study that identified Klotho as an essential effector for the antifibrotic effects of HDAC3 knockdown or pharmacological inhibition in mouse models of renal fibrosis (*113*, *114*). Beyond the renal system, previously unidentified roles for HDAC3 have also been uncovered in the intestinal context.

#### Intestinal homeostasis

Discoveries have revealed an emerging role for HDAC3 in intestinal homeostasis. Commensal intestinal bacteria were found to produce inositol-1,4,5-trisphosphate, which supports HDAC3 interaction with NCoR1/2, and its deacetylase activity (*115*). Thereby, commensal bacteria were found to stimulate HDAC3 activity, which promoted recovery following intestinal damage, identifying HDAC3 as a sensor of metabolites that calibrates host response to microbes (*115*). HDAC3 was found to regulate local microbiota-specific immunity, as epithelial deletion of HDAC3 resulted in accumulation of commensal-specific CD4^+^ T cells in the intestine (*116*). Aberrant immune response to resident microbes promotes inflammatory bowel disease and other chronic inflammatory conditions and, correspondingly, HDAC3 was implicated in preventing T cell–driven colitis (*116*). Further exploration of the cross-talk between microbiota and intestinal epithelia uncovered a role for HDAC3 in stem cells for tuft cell differentiation that was dampened by a commensal metabolite, implicating HDAC3 in intestinal type 2 immunity, which protects from parasitic infection (*117*).

#### Epidermal development

In epidermal development, HDAC3 was identified as a hub that coordinates orderly stepwise epidermal stratification (*118*). Comparing HDAC3 and NCoR1/2 in vivo knockout phenotypes revealed that, while HDAC3 function requires NCoR1/2, in the epidermis, its mechanism of action is largely independent of HDAC activity (*118*). Instead, HDAC3 was found to cooperate with transcription factor Kruppel-like factor 4 (KLF4) to repress *TGM1*, *KRT16*, and *AQP3* gene expression (*118*). These findings provide a previously unidentified biological context where HDAC3 partners with tissue-specific transcription factors to drive biological function.

#### Bone

HDAC3 also coordinates transcription factor networks in bone development and disease, and new findings have also been uncovered in this tissue context. In bone development, receptor activator of nuclear factor kappa-B (RANK) is required for the development of osteoclasts from myeloid progenitor cells (*119*). Findings uncovered that RANK signaling converts the NCoR/HDAC3 corepressor complex to a coactivator of AP-1 and NF-κB target genes required for osteoclast differentiation (*119*). RANK signaling promoted the assembly of an NCoR/HDAC3/PPARγ coactivator 1-beta (PGC1β) complex that was dependent on noncoding RNAs, resulting in the conversion of NCoR from a constitutive corepressor to a signal-dependent coactivator of gene expression (*119*). Intriguingly, these findings support the concept of NCoR/HDAC3 complexes as signal-dependent coactivators, challenging the corepressor paradigm (*119*). A separate study of cartilage stiffness uncovered HDAC3 as a mechanosensitive mechanism affecting chondrocyte physiology (*120*). ECM stiffening is a characteristic of cartilage aging, which is a feature of osteoarthritis (OA) (*120*). ECM stiffening was found to accelerate chondrocyte senescence and down-regulate HDAC3, whereas intra-articular injection of HDAC3-expressing adeno-associated virus restored the young phenotype of aged chondrocytes stimulated by ECM stiffening and alleviated OA (*120*). Together, studies in bone have revealed both previously unidentified mechanistic paradigms and biology regulated by HDAC3. Complementary mechanistic insights have also been reported in macrophages.

#### Macrophages

Studies in macrophages have revealed key insights into HDAC3 mechanism of action, revealing that dynamic interplay with multiple transcription factor programs guides and directs distinct HDAC3-driven biology. Analogously to findings from osteoclast differentiation described above (*119*), a coactivator role for the PGC1β-containing HDAC3/NCoR complex was also identified in macrophages (*121*). Toll-like receptor 4 signaling promoted assembly of an HDAC3/NCoR/PGC1β coactivator complex and HDAC3-driven deacetylation and activation of PGC1β (*121*). TNF receptor–associated factor 6 (TRAF6) was implicated as an upstream effector regulating the conversion of the NCoR/HDAC3 corepressor complex to a signal-specific HDAC3/NCoR/PGC1β coactivator complex required for inflammatory gene activation in macrophages (*121*). In a separate study investigating how global loss of HDAC3 resulted in repression of gene expression, activation of macrophages by lipopolysaccharides (LPS) was found to recruit HDAC3 to ATF2-bound genomic sites without NCoR1/2 (*122*). HDAC3 cooperation with ATF2 resulted in expression of inflammatory genes, whereas HDAC3 deacetylase activity was engaged at ATF3-bound sites that suppressed Toll-like receptor signaling (*122*). Furthermore, loss of HDAC3 in macrophages safeguarded mice from lethal exposure to LPS, but this was not conferred upon abolition of HDAC3 catalytic activity (*122*). IFN-α/β activates the JAK/STAT signaling pathway and suppresses viral replication through the induction of IFN-stimulated genes (*123*). A study identified that HDAC3 in macrophages maintains expression of signal transducer and activator of transcription 1 and 2 (STAT1/2), and inactivation of HDAC3 leads to defective antiviral immunity (*123*). Regulation of STAT1/2 was attributed to HDAC3 cooperation with the transcription factor Forkhead Box K1 (FOXK1), and HDAC3 was found to protect FOXK1 from degradation (*123*). Alveolar macrophages (AMs), the resident macrophages in lung alveoli, contribute to the maintenance of airway homeostasis and are implicated in multiple pulmonary diseases (*124*). Findings demonstrated that HDAC3 was required for AM embryonic development, postnatal homeostasis, maturation, and regeneration from bone marrow (*124*). Loss of HDAC3 resulted in mitochondrial oxidative dysfunction attributed to HDAC3 regulation of PPARγ expression and pathway activity (*124*). While macrophages have served as a valuable conduit for enhancing the understanding of transcription factor networks regulated by HDAC3, findings from the T cell context have continued to define the biology regulated by HDAC3.

#### T cells

Findings implicate HDAC3 in cytotoxic T cell and regulatory T cell function. Following infection, naïve CD8 T cells become activated and develop into effector cytotoxic T lymphocytes that mediate immunity by killing infected cells (*125*). Findings identified that HDAC3 inhibits CD8 T cell cytotoxicity early during activation and is required for persistence of activated CD8 T cells following resolution of an acute infection (*125*). HDAC3 was found to regulate gene programs associated with cytotoxicity and effector differentiation of CD8 T cells, including essential cytotoxicity genes and key transcription factors (*125*). T_regs_ specifically express the master transcription factor FOXP3 and have a key role in immunological tolerance and homeostasis (*126*). Disruption of T_reg_ function leads to autoimmune disease, motivating discovery of means to enhance T_reg_ function and stability (*126*). A whole-genome CRISPR screen in primary human T cells was used to uncover regulators of FOXP3 induction, and findings were validated by coupling pooled CRISPR knockouts with single-cell RNA readouts (Perturb-icCITE-seq) (*126*). This approach uncovered that the RBPJ-NCOR complex represses *FOXP3* expression via HDAC3-driven histone deacetylation, revealing the HDAC3 complex as a target for improving efficacy of adoptive cell therapy for autoimmune disease (*126*). Immune cells including macrophages and T cells are critical in cancer; it follows that previously unidentified roles for HDAC3 have also been identified in cancer.

#### Cancer

Findings on the roles of HDAC3 in cancer suggest that inhibition of HDAC3 is a promising approach across a breadth of cancer types including blood cancer, breast cancer, CRC, lung cancer, pancreatic cancer, and other cancers as well.

#### Blood cancer

Multiple groups have reported roles for HDAC3 in blood cancer including leukemia, lymphoma, and myeloma. In AML, dependency on HDAC3 has been uncovered in multiple subsets of the disease driven by different oncogenic rearrangements, suggesting that targeting HDAC3 may be more broadly relevant than previously appreciated. In a study of the AML fusion oncoprotein DEK::NUP214, DEK was found to recruit the NCoR1/HDAC3 complex to repress H3K27ac and restrict the chromatin accessibility of hematopoietic stem cells, governing the expression of quiescence-associated genes (*127*). AML with *RARG* fusions was identified as a previously unidentified subtype with poor clinical outcomes (*128*). A study of the most prevalent *RARG* fusion, *CPSF6::RARG*, found that fusion protein interaction with HDAC3 suppressed expression of myeloid differentiation genes including *PU.1*, and targeting this axis via HDACi suppressed leukemia in vivo (*128*). In *PML::RARα*-driven APL, HDAC3 inhibition was identified as a means to degrade the PML::RARα oncoprotein (*129*). Another study identified that proline-199 (P199) of the transcription factor ERG is causal to ERG’s ability to induce leukemia (*130*). P199 was found to facilitate ERG’s interaction with the NCoR/HDAC3 complex, which revealed HDAC3 inhibition as a means to reduce growth of ERG-dependent cancers (*130*).

An aggressive form of B cell lymphoma carries somatic mutations in the gene *TBL1XR1* (*131*). Findings uncovered that *TBL1XR1* mutations skew the humoral immune response toward generating abnormal immature memory B cells while impairing plasma cell differentiation (*131*). TBL1XR1 mutants were found to promote NCoR2/HDAC3 toward binding with the memory B cell transcription factor BACH2 at the expense of binding the germinal center (GC) transcription factor BCL6, leading to pre-memory transcriptional reprogramming and cell-fate bias (*131*). TBL1XR1 alterations led to a notable extranodal immunoblastic lymphoma phenotype that mimicked human disease (*131*), providing evidence that observed mutations in TBL1XR1 are causal to disease. A study of the H3K27 demethylase lysine demethylase 6A (KDM6A) in multiple myeloma (MM) reported that HDAC3 inhibition with RGFP966 increased MHC expression, implicating HDAC3 inhibition as a means to restore immunogenicity (*132*). HDAC3 has emerged as a promising target in blood cancer, and it will be interesting to learn the extent to which this holds true across additional subsets of blood cancer. As in MM, targeting HDAC3-containing complexes to restore antitumor immunity has also been identified as a promising approach in breast cancer.

#### Breast cancer

The well-established model systems in the breast cancer field have revealed insights into HDAC3 function in cancer. A functional proteomic screen of organoids derived from chemotherapy-treated patients with breast cancer identified NCoR2 as an inhibitor of cytotoxic stress response and antitumor immunity (*133*). NCoR2 was found to inhibit antitumor treatment by regulating HDAC3 to repress interferon regulatory factor 1 (IRF-1)–dependent gene expression and IFN signaling (*133*). Reducing NCoR2 or modifying its interaction with HDAC3 was sufficient to enhance chemotherapy responsiveness and restore antitumor immunity (*133*). Separately, HDAC3 activity in the soft niches of breast tumors was implicated in up-regulation of gene expression associated with brain metastasis, and HDAC3 inhibition with RGFP966 reduced niche softness-induced brain metastatic ability (*134*). HDAC3 as a critical regulator of inflammatory response in cancer has also been characterized in CRC.

#### Colorectal cancer

In the context of CRC, findings implicate HDAC3 in regulation of gene expression programs that affect tumor immunity. Microsatellite stable (MSS) CRCs are often associated with resistance to anti–PD-1 therapy, and a study found that the CRC pathogen *Fusobacterium nucleatum* (*Fn*) sensitizes MSS CRC to anti–PD-1 (*135*). Intratumoral *Fn* was linked to production of butyric acid, which was associated with inhibition of HDAC3 and HDAC8 activity but increased expression of T-box transcription factor 21 (*Tbx21*) in CD8^+^ T cells; TBX21 regulated efficacy of anti–PD-1 by transcriptionally repressing PD-1 (*135*). A study of homeodomain-interacting protein kinase 2 (HIPK2) in inflammatory macrophages implicated phosphorylation of HDAC3 as a means of regulating NF-κB p65 acetylation at lysine-218 to control its activation (*136*). Findings implicate this pathway as a means to restrain inflammation in colitis-associated CRC and sepsis (*136*). A study exploring the impact of ECM stiffness on the CD8^+^ T cells identified that the transcription factor OSR2 integrates biomechanical signaling and facilitates the terminal exhaustion of tumor-reactive CD8^+^ T cells (*137*). Depletion of OSR2 alleviated exhaustion of tumor-specific CD8^+^ T cells or CAR-T cells in colon cancer cell models (*137*). Recruitment of HDAC3 was implicated in the ability of OSR2 to suppress cytotoxic gene expression and promote CD8^+^ T cell exhaustion (*137*). In contrast, HDAC3 was found to regulate both the TME and tumor cell intrinsic mechanisms in lung cancer.

#### Lung cancer

HDAC3 was found to regulate key gene expression programs in KRAS mutant lung cancer. GEMMs revealed an essential role for HDAC3 in *Kras, Trp53* and *Kras, Lkb1* mutant LUAD (*138*). HDAC3 and NKX2-1 were found to coordinately regulate fibroblast growth factor receptor 1 (*Fgfr1)* expression in an LKB1-dependent manner (*138*). Furthermore, an HDAC3-dependent transcriptional cassette became hyperactivated upon cellular resistance to the mitogen-activated protein kinase kinase (MEK) inhibitor trametinib, and combination treatment of trametinib with the HDAC1/HDAC3 inhibitor entinostat elicited therapeutic benefit in the *Kras, Lkb1* mutant GEMM (*138*). In a subsequent study, genetic HDAC3 inactivation selectively within lung tumor cells resulted in T cell influx into both *Kras, Trp53* and *Kras, Lkb1* mutant GEMM tumors (*139*). This phenotype was ascribed to direct repression by HDAC3 of a group of chemokine genes including *Cxcl10*, and combination with KRAS pathway inhibitors further enhanced immunosurveillance-related chemokine gene expression (*139*). T cell recruitment into tumors could be elicited by combination treatment using clinically tolerated targeted therapies, and recruited T cells contributed to tumor growth control (*139*). Findings reveal a previously unidentified targeted therapy approach for the immune-cold *Kras, Lkb1* mutant subtype of LUAD that is refractory to standard-of-care therapy, and findings suggest that targeting HDAC3 may provide means to simultaneously restrain tumor growth and enhance immune-mediated tumor clearance of *KRAS* mutant LUAD.

#### Pancreatic cancer

In pancreatic cancer, combinatorial treatment strategies that target HDAC3 have also been identified as promising approaches. A study of molecular mechanisms underlying targeted therapy resistance in pancreatic ductal adenocarcinoma (PDAC) identified the epigenetic factor SETD5 as a major driver of resistance to MEK inhibition (*140*). SETD5 scaffolds a corepressor complex including HDAC3 and histone methyltransferase G9a, and pharmacological cotargeting of MEK, HDAC3, and G9a was found to sustain PDAC tumor growth inhibition in vivo (*140*). Separately, class I HDACs were found to facilitate the induction of prodesmoplastic and protumorigenic transcriptional programs in pancreatic stromal fibroblasts (*141*). HDAC depletion in cancer-associated fibroblasts and treatment with the HDAC inhibitor entinostat in PDAC mouse models reduce stromal activation and curbed tumor progression (*141*).

#### Other cancers

HDAC3 has also been implicated in other cancer types including melanoma, bladder cancer, HCC, and prostate cancer. Findings from melanoma and bladder cancer report HDAC3 targeting as a means to overcome ICI resistance. In melanoma, the IκBζ coregulator of NF-κB was found to suppress expression of *Cxcl9*, *Cxcl10*, and *Ccl5* via HDAC3 and EZH2 to impair the recruitment of NK and CD8^+^ T cells into the tumor, causing resistance to α-PD-1 immunotherapy in mice (*142*). In bladder cancer, entinostat promoted immune editing of tumor neoantigens, remodeled the tumor immune microenvironment, and induced an antitumor response (*143*). Combination treatment with anti–PD-1 and entinostat led to complete responses and conferred long-term immunologic memory, providing preclinical rationale for combined entinostat and PD-1 blockade in bladder cancer (*143*).

In HCC, hepatocyte-specific deletion of HDAC3 was shown to promote HCC tumorigenesis in female mice but not male mice (*144*). Loss of HDAC3 reduced Forkhead box A1/A2 (*FOXA1/2)* expression and induced estrogen-dependent tumorigenesis in HDAC3-ablated livers of female mice (*144*).

In prostate cancer, HDAC3 was found to interact with the homeodomain transcription factor HOXB13, which is disrupted by the HOXB13 Gly84→Glu (G84E) mutation that has been associated with early-onset prostate cancer (*145*). Independently of AR, HOXB13 was found to recruit HDAC3 to suppress lipogenic gene expression including fatty acid synthase, thereby restraining cell motility and xenograft tumor metastasis (*145*). In contrast to findings from other cancer types that identify HDAC3 as a druggable cancer dependency, this study suggests that, in prostate cancer, HDAC3 may restrain tumor growth.

Together, these findings suggest that HDAC3 may determine both tumor-intrinsic growth properties and tumor immunity across a broad range of cancers. Accumulating evidence suggests that aberrant activity and/or expression of HDAC3 interacting proteins enhances function of HDAC3 multiprotein complexes to drive resistance and progression in a breadth of cancer types. However, distinct cancer types appear to use distinct protein partners to impart aberrant HDAC3 activity. Findings suggest remarkably nonredundant roles of HDAC3 for which other HDAC isoforms cannot compensate, positioning HDAC3 as a particularly promising drug target for the treatment of cancer. Clinically viable HDAC3-selective inhibitors will be critical additions to the cancer pharmacopeia.

Collectively, findings across systems suggest a role for HDAC3 that includes and extends beyond serving as a canonical repressor of gene expression. These findings support the concept of HDAC3 as a signal-responsive conduit for orchestrating nuanced gene expression programs. A variety of molecular signals have emerged as regulators of HDAC3 programs, including metabolites produced by regional bacteria. In addition, PGC1β association with the NCoR/HDAC3 complex has been shown to transform the corepressor into a coactivator complex. Mechanisms of gene regulation beyond canonical histone deacetylation continue to be defined for HDAC3, and findings have enhanced the understanding of transcription factor and coregulator network interplay that determines HDAC3 control of gene expression in physiology and disease. Increased awareness of HDAC3’s interactome provides previously unidentified understanding of context-specific HDAC3 dependencies, which could facilitate development of previously unknown approaches for precision targeting of HDAC3 with enhanced therapeutic efficacy and minimized on-target toxicities.

### HDAC8

Although less studied than the other class I HDACs, previously unidentified roles for HDAC8 in gene expression control have been identified in the nervous system, intestinal homeostasis, pulmonary fibrosis, and cancer (Fig. 5). In peripheral Schwann cells of the nervous system, HDAC8 ablation was found to accelerate the regrowth of sensory axons and enhance recovery after nerve injury (*146*). HDAC8 recruited the E3 ubiquitin ligase TRAF7 to degrade HIF-1α, resulting in the down-regulation of *c-Jun*, an inducer of Schwann cell–driven nerve repair (*146*). Because the molecular mechanism identified was independent of HDAC8 catalytic function, HDAC8 inhibitors targeting the catalytic domain were not expected to promote nerve repair (*146*). Instead, promising HDAC8-targeting strategies would need to reduce total HDAC8 protein levels or disrupt the HDAC8/TRAF7 interaction (*146*).

Microbially derived butyrate, a short-chain fatty acid commonly found in the intestine, was found to promote intestinal homeostasis via repression of the glycolytic enzyme hexokinase 2 (HK2) (*147*). Intestinal epithelial cell–specific knockout mouse and human organoid models were used to define the role of HK2 function in pathological intestinal inflammation (*147*). Butyrate-driven reduction in *HK2* gene expression could be prevented by cotreatment with the HDAC8 inhibitor PCI-34051, implicating HDAC8 as a potential molecular intermediate between butyrate and HK2 (*147*). Findings suggest HK2 inhibition as a potential therapeutic approach for intestinal inflammation disorders (*147*).

In the context of pulmonary fibrosis, findings suggest that HDAC8 activation rather than inhibition could provide therapeutic benefit. Protein levels of pleckstrin homology domain and leucine-rich repeat protein phosphatase 1 (PHLPP1) were observed to decline in pulmonary fibrosis (*148*). Using AM-specific deletion in vivo, PHLPP1 was found to protect against lung fibrosis by repressing the *KLF4* gene expression program (*148*). PHLPP1 was identified to directly interact with and dephosphorylate HDAC8 at Ser^39^, resulting in increased HDAC8-mediated histone deacetylation and repression of the *KLF4* gene program (*148*). Findings suggest that activation of HDAC8 would be required for eliciting desired impact on disease.

Roles for HDAC8 in cancer have also begun to emerge. Combination HDAC8 inhibition together with replication checkpoint kinase inhibition was found to be synthetically lethal in cancer cells (*149*). HDAC8 reduced acetylation of structural maintenance of chromosomes protein 3 (SMC3) ahead of replication forks, and HDAC8 inactivation resulted in slowed fork progression (*149*). Findings implicated HDAC8 as a means to impinge upon genome integrity and leverage cancer-specific sensitivity to replication stress as a therapeutic vulnerability (*149*). Separately, using cardiac injection of melanoma cells, HDAC8 expression was found to enhance brain metastasis but not liver or lung metastasis (*150*). The enhanced invasiveness of cells was attributed to downstream function of E1A binding protein p300 (EP300) (*150*). As in nerve repair, in B-ALL leukemia, HDAC8 was also found to regulate degradation of HIF-1α (*151*). Selective HDAC8 inhibition using inhibitor 22d slowed leukemia progression in combination with tyrosine kinase inhibition (*151*).

Together, recent discoveries have augmented understanding of disease systems involving known HDAC8 substrates like SMC3 and identified multiple molecular mechanisms through which HDAC8 regulates HIF-1α. Studies suggest therapeutic benefit could be achieved by inhibiting or activating HDAC8 depending on the disease context. Future therapeutic strategies targeting HDAC8 will need to remain cognizant of these context-specific nuances.

### Class IIa HDACs: HDAC4, HDAC5, HDAC7, and HDAC9

The class IIa HDACs comprise HDAC4, HDAC5, HDAC7, and HDAC9, and each contains a highly conserved deacetylase domain in their C terminus (*4*, *5*). A conserved substitution of a key catalytic tyrosine residue to histidine within the catalytic domain of the class IIa HDACs accounts for their drastically reduced catalytic activity compared to class I HDACs (*152*). Instead, the class IIa HDACs are characterized by an extended N-terminal adapter domain that contains regulatory phosphorylation sites and protein binding domains. These include a well-characterized binding domain for the transcription factor myocyte enhancer factor 2 (MEF2), and class IIa HDAC association with MEF2 represses expression of its target gene program (*5*). The N-terminal domain also contains phosphorylation sites that regulate association with the chaperone protein 14-3-3 (*4*, *5*). When unphosphorylated, the class IIa HDACs are nuclear, whereas phosphorylation results in shuttling of the class IIa HDACs into the cytoplasm (*5*).

**Fig. 6.**
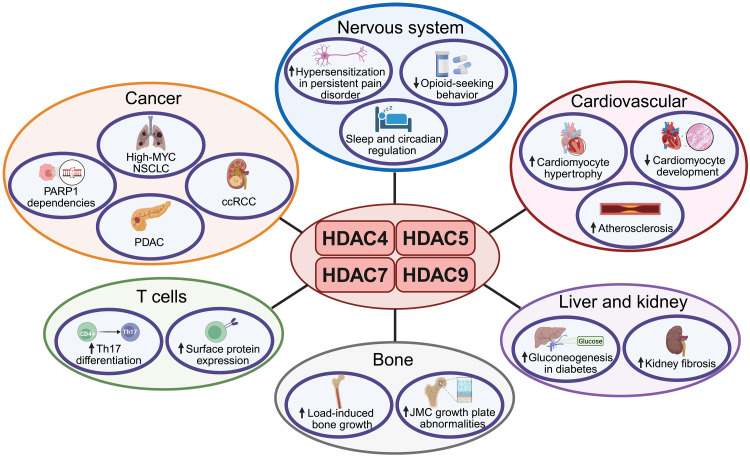
Findings define previously unidentified roles for class IIa HDACs in physiology and disease. Arrows indicate directionality associated with class IIa HDAC activity. Created in BioRender. Van Bree, B. (2026). https://BioRender.com/lxpnmdj.

Physiologically, class IIa HDACs have restricted expression patterns in vivo (*5*). Original reports identified high expression of HDAC4 in brain, skeletal growth plate, liver, and muscle tissue; HDAC5 expression in muscle, heart, liver, and brain tissue; HDAC7 expression in ECs, thymocytes, liver, muscle, and heart tissue; and HDAC9 expression in muscle, heart, brain, and adipose tissue (*4*, *5*, *153*, *154*). Roles of the class IIa HDACs in health and disease remain to be fully characterized, and findings implicate the gene expression regulatory roles of class IIa HDACs across a breadth of normal physiology and disease states. Previously unidentified roles for class IIa HDACs continue to be defined in tissues where original genetic models identified developmental phenotypes, and new contexts continue to be uncovered where class IIa HDACs are essential. Emerging areas of focus discussed here include the nervous system, cardiovascular system, liver and kidney, bone, T cells, and cancer (Fig. 6). Because class IIa HDACs are functionally redundant in vivo (*5*, *153*), here we summarize findings about class IIa HDACs together but delineate the roles of each isoform. Collectively, these findings augment understanding about HDAC subfamily- and isoform-specific roles in regulating gene expression that will be essential for the development of class IIa HDAC targeted therapies with enhanced therapeutic efficacy and minimal on-target toxicity.

#### Nervous system

In the nervous system, HDAC4 and HDAC5 were implicated in regulation of sleep, addiction, and pain. Using an adult mouse brain knockout system, HDAC4 and HDAC5 were found to regulate the amount of non–rapid eye movement sleep (NREMS) and delta power. HDAC4 regulation of NREMS amount was localized to the posterior hypothalamus, and the related regulation of gene expression was linked to HDAC4 cooperation with CREB (*155*). A separate study also identified that loss of HDAC4 and HDAC5 increased NREMS in mice (*156*). Both studies implicated the LKB1-Salt-inducible kinase 3 (SIK3) pathway as a key upstream regulatory mechanism via direct phosphorylation of HDAC4/HDAC5 (*155*, *156*). This second study extensively explored LKB1-SIK3 signaling control of HDAC4 impact on sleep and wakefulness with neural-specific gain- and loss-of-function approaches in mice, linking the LKB1-SIK3-HDAC4 molecular pathway and its target genes with mammalian sleep need (*156*). Cycles of sleep and wakefulness in mammals is governed by the suprachiasmatic nucleus (SCN), a structure in the hypothalamus considered the central pacemaker of circadian rhythmicity (*157*). The SIK3-HDAC4 pathway in the SCN, composed of GABAergic neurons, was found to regulate the circadian period length and the timing of arousal without affecting total sleep amount and depth (*157*). Together, the LKB1-SIK3-HDAC4 molecular axis has emerged as a major effector of sleep architecture, with distinct roles in specific neurons.

Consistent with key molecular effectors in health being disrupted in disease states, roles for HDAC4 and HDAC5 have also been identified in opioid-seeking behavior and pain disorders. Opioid use produces associations between drug reinforcement and the drug-taking environment, and these associations can trigger relapse in individuals recovering from opioid use disorder (*158*). In an animal model of opioid use disorder, roles for HDAC5 in limiting distinct triggers of drug-seeking behavior were identified in both dopamine D1 and D2 receptor-expressing medium spiny neurons during active heroin use (*158*). Findings suggest that HDAC5 may limit relapse vulnerability through regulation of ion channel gene expression and suppression of medium spiny neuron firing rates (*158*). Chronic pain is a debilitating condition that is considered a pathological manifestation of neuronal plasticity in nociceptive pathways (*159*). In mouse models of persistent inflammatory pain disorders, HDAC4 was identified as a key regulator that translated nociceptive activity into neuronal hypersensitization by regulating gene expression of organic anion transporter 1 (*OAT1*) (*159*). The study implicated the HDAC4-OAT1 pathway as a potential avenue for pain-relieving therapies.

Where LKB1-SIK signaling potently dictates class IIa HDAC activity, targeting upstream kinases may provide therapeutic opportunities for drugging the class IIa HDACs in disease states. As in the nervous system, SIKs have also been implicated as key regulators of class IIa HDACs in the cardiovascular system.

#### Cardiovascular system

Although roles for class IIa HDACs in cardiac function are well established, discoveries have revealed previously unidentified roles for class IIa HDACs in cardiovascular system function and pathology. In cardiac reprogramming, HDAC4 was reported to organize nuclear condensates that inhibit gene programs responsible for cardiomyocyte formation from fibroblasts in vitro (*160*). Disruption of HDAC4-organized condensates promoted cardiac gene expression (*160*). A role for SIK1 in cardiac remodeling and heart failure pathogenesis was identified using rodent models and cardiomyocytes derived from human induced pluripotent stem cells (*161*). In this system, HDAC7 was identified as a key prohypertrophic signaling effector of SIK1, and indirect induction of the c-MYC transcriptional program was implicated as a downstream mechanism of action (*161*). Thus, the SIK1-HDAC7 molecular axis was identified as a potential target for heart failure (*161*). Endothelial-to-mesenchymal transition is associated with atherosclerosis; however, the molecular pathways governing this process are poorly defined (*162*). HDAC9 was implicated in atherosclerosis pathology; in vitro and in vivo evidence suggested a pathological link between endothelial-to-mesenchymal transition, HDAC9, and atherosclerosis (*162*). Therefore, targeting HDAC9 may be beneficial for plaque stabilization or slowing atherosclerotic progression (*162*).

#### Liver and kidney

Previously unidentified roles for class IIa HDACs have been uncovered in the liver and kidneys. In the liver, HDAC7 activity mediates a pathway for negatively regulating hepatic gluconeogenesis in mouse models relevant to diabetes (*163*). Mitochondrial β-oxidation enzyme HADHA was reported to promote β-HB production and negatively regulate murine hepatic gluconeogenesis during glucagon challenge by targeting HDAC7 (*163*). HDAC9 was reported to be an attractive therapeutic target for kidney fibrosis (*164*). Renal tubule-specific HDAC9 deletion or inhibition in male mice was found to attenuate epithelial cell cycle arrest, reduced production of profibrotic cytokines, and alleviated tubulointerstitial fibrosis (*164*).

#### Bone

Class IIa HDACs have also been implicated in the regulation of gene expression in osteocytes and chondrocytes. Osteocytes, which are the primary skeletal mechanosensors, sense mechanical cues. Loading-induced reduction in osteocytic sclerostin (*SOST*) expression stimulates bone formation. Findings identified that HDAC4 and HDAC5 are required for load-induced *SOST* suppression and bone formation (*165*). Focal adhesion kinase (FAK) was implicated as an upstream regulator of HDAC5 phosphorylation and subcellular localization that links matrix-derived cues to HDAC5 regulation of *SOST* gene expression (*165*). Jansen’s metaphyseal chondrodysplasia (JMC) is a rare disorder caused by activating mutations in parathyroid hormone receptors that presents with bone growth plate abnormalities. Findings from a humanized mouse model of JMC identified that *HDAC4* genetic ablation rescued the growth plate abnormalities characteristic of JMC that led to abnormalities in skeletal development and mineral ion homeostasis (*166*).

#### T cells

In a study on T cell differentiation, HDAC4 and HDAC7 were identified to guide the lineage-specific differentiation of mouse T_H_17 cells from naive CD4^+^ T cells (*167*). HDAC4 was found to drive expression of T_H_17 signature genes including *IL17A/F*, whereas HDAC7 repressed expression of T_H_17 negative regulators including *IL2*, and class IIa HDACi mitigated T_H_17 cell–mediated intestinal inflammation in a colitis model (*167*). Separately, a study of alternative splicing identified that a long splice isoform of HDAC7 in T cells controlled its interaction with protein chaperons and promoted expression of several T cell surface proteins including CD3, CD28, and CD69 (*168*).

#### Cancer

The role of the class IIa HDACs in cancer remains to be clearly elucidated, but evidence continues to emerge linking the class IIa HDACs to a variety of cancer processes. Two studies identified a link between class IIa HDACs and the DNA repair protein PARP1 (*169*, *170*). Direct interaction between HDAC5 and PARP1 was identified to regulate expression of target genes (*169*), and SIK2 inhibition was identified as a means to regulate class IIa HDAC target gene expression and sensitize ovarian and TNBC cells to PARP inhibition in xenograft models (*170*). Another study identified a noncanonical delactylase role for HDAC5 in sensitizing TNBC to radiotherapy with saikosaponin D (SSD) treatment (*171*). SSD treatment increased expression of HDAC5 and increased delactylation of the DNA repair protein MRE11, and SSD cotreatment with irradiation elicited an antitumor effect (*171*). In clear cell renal carcinoma (ccRCC), HDAC7 was identified to support tumor growth in vivo, and findings were linked with regulation of a branched-chain amino acid catabolism gene expression program (*172*). A study aimed at discovering combinatorial strategies for enhancing the therapeutic efficacy of targeting MYC in lung cancer identified HDAC5 and HDAC9 as promising targets, and the antitumor impact of dual MYC and class IIa HDAC targeting was associated with elevated mitochondrial reactive oxygen species (*173*). Separately, a gain-of-function screen for epigenetic regulators in the *Kras*^G12D^; *Trp53*^−/−^ PDAC mouse model identified that HDAC5 enables *KRAS* mutant-independent tumor growth (*174*). HDAC5 control of *Socs3* regulated *Ccl2* gene expression, which affected macrophage recruitment to tumors, thereby uncovering a means through which HDAC5 potentiates tumor growth in the absence of KRAS expression (*174*).

Phosphorylation has continued to emerge as a dominant upstream regulator of the pleiotropic impacts of the class IIa HDACs on gene expression across biological contexts. The well-conserved biochemical link between signaling and the class IIa HDACs may provide a promising opportunity for targeting class IIa HDAC-dependent disease. Whether therapeutic strategies target upstream signaling effectors or the class IIa HDACs directly, treatments that consider specific HDAC subfamily or isoform vulnerabilities in disease pathways are most likely to overcome the barriers to translational success that have plagued the HDAC field.

### Class IIb HDACs: HDAC6 and HDAC10

**Fig. 7.**
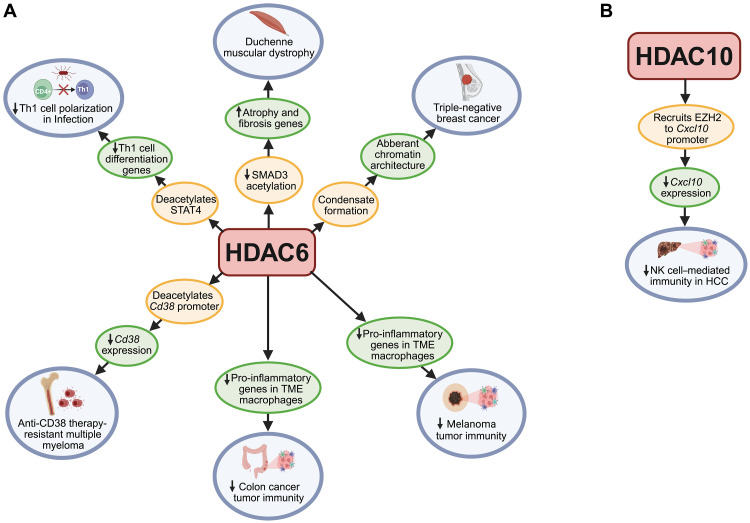
Findings define previously unidentified roles for HDAC6 and HDAC10. Arrows indicate directionality associated with (**A**) HDAC6 activity and (**B**) HDAC10 activity. Created in BioRender. Van Bree, B. (2026). https://BioRender.com/t72ybj2.

The class IIb HDACs comprise HDAC6 and HDAC10. Both class IIb HDACs contain a C-terminal extension, the tail domain. They are also typically localized to the cytoplasm, although HDAC10 has been suggested to have both nuclear and cytoplasmic roles (*175*). HDAC6 is established to deacetylate several cytoplasmic substrates including α-tubulin (*4*, *176*–*180*), and HDAC10 has been comparatively less studied than HDAC6. As cytoplasmic enzymes, the class IIb HDACs do not primarily regulate gene expression (*181*). Nonetheless, studies identified roles for the class IIb HDACs as regulators of gene expression programs in muscle, response to infection, and cancer (Fig. 7). Cognizance of the nonredundant roles of the class IIb HDACs will augment future therapeutic strategies for overcoming class IIb HDAC-dependent disease states.

In the *mdx* mouse model of Duchenne muscular dystrophy, the HDAC6 inhibitor tubastatin A reversed muscular dystrophy phenotypes by increasing muscle strength and reducing muscle atrophy and fibrosis (*182*). Reduced dystrophy-associated gene expression observed upon HDAC6 inhibition was attributed to acetylation of transcription factor (*182*).

In response to *Listeria* infection, HDAC6-deleted mice exhibited elevated T_H_1 cell differentiation and decreased infection severity (*183*). Findings were attributed to lysine-667 acetylation of STAT4 required for the T_H_1 cell differentiation gene expression program (*183*).

Advances in the study of HDAC6 in cancer have been potentiated by the development of HDAC6 inhibitors with improved safety profiles. Findings have identified promise for combining HDAC6 inhibitors with immunotherapies and identified previously unidentified roles for HDAC6 in cell biology and chromatin architecture. HDAC6 inhibition has been established to enhance the antitumor immune response and reduce M2 macrophage polarization in several cancers including colon cancer and melanoma (*184*). In contrast to multiple HDAC6 inhibitors whose structure precludes clinical application, AVS100 (SS208) was identified to have a desirable safety profile and was approved for clinical use (*184*). Findings in melanoma and colon cancer models identified that AVS100 increases the efficacy of anti–programmed cell death protein 1 (PD-1) treatment, implying that the combination of pembrolizumab with HDAC6i could be a promising approach for locally advanced or metastatic solid tumors (*184*). In MM, daratumumab is an approved anti-CD38 antibody therapy (*185*). During therapy, expression of the transmembrane glycoprotein CD38 on MM cells declines, favoring immune escape and disease progression (*185*). Predicated on the concept that HDAC6 inhibition could increase CD38 expression, the HDAC6-targeting drug ricolinostat was found to increase sensitivity to daratumumab, and additional compounds targeting HDAC6 were also identified (*185*). Separately, phosphorylated HDAC6 was found to form liquid-liquid phase separation condensates in nuclei of TNBC cells, and disrupting these condensates suppressed tumor growth (*186*). The phospho-HDAC6–induced aberrant chromatin architecture was found to affect chromatin accessibility, histone acetylation, RNA polymerase II elongation, and transcriptional profiles (*186*).

Previously unidentified roles for HDAC10 in cancer have also been identified. In HCC, EZH2 was found to directly repress expression of CXCL10 in an HDAC10-dependent manner (*187*). CXCL10 was found to be necessary and sufficient for stimulating NK cell migration, and CXCL10 was implicated in the tumor growth control response to EZH2 inhibition (*187*).

### Class IV HDAC: HDAC11

**Fig. 8.**
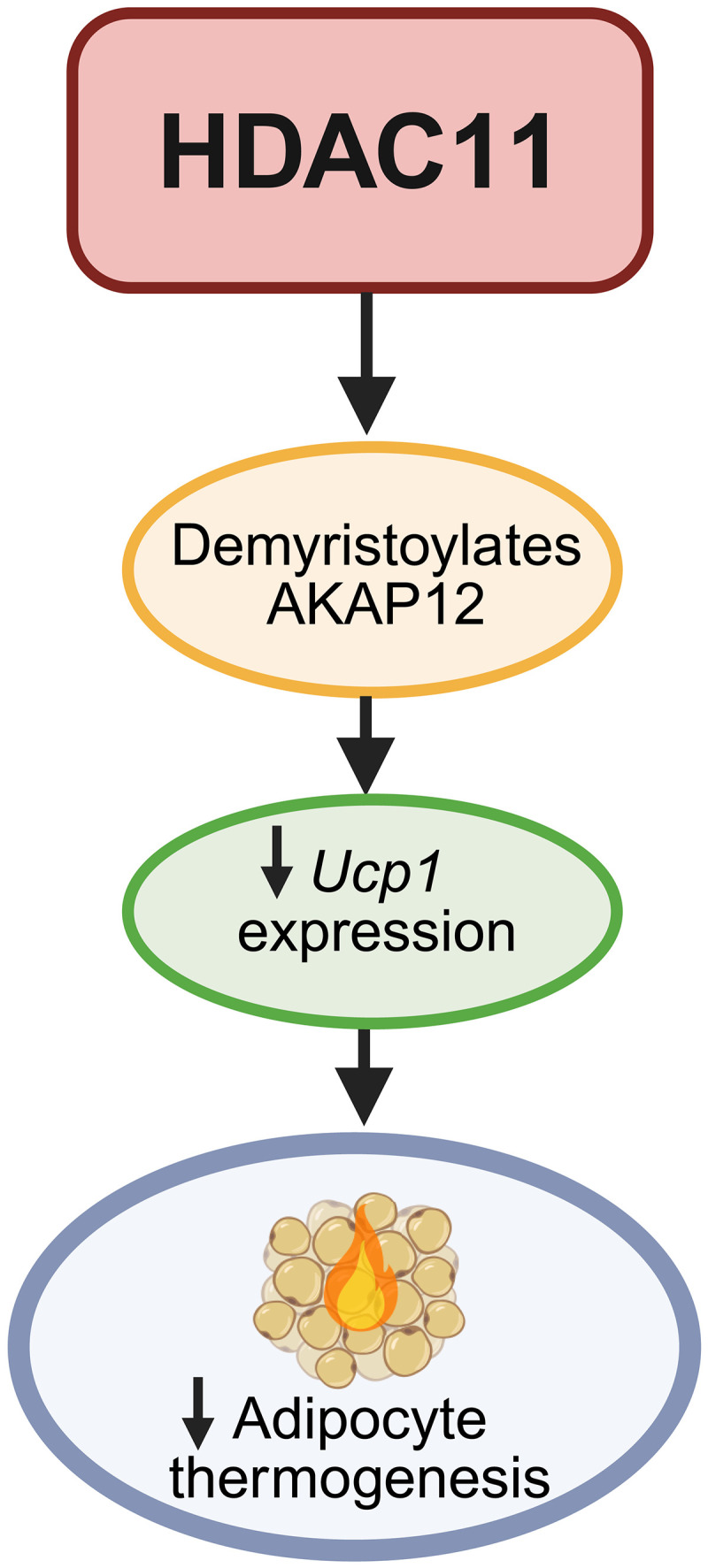
Previously unidentified role for HDAC11 in adipocytes. Arrows indicate directionality associated with HDAC11 activity. Created in BioRender. Van Bree, B. (2026). https://BioRender.com/vhv3m3l.

HDAC11 is the sole member of the class IV HDACs, and the least studied of the zinc-dependent HDAC protein family. HDAC11 primarily localizes to the nucleus, and it has been shown to exhibit HDAC activity in vitro (*17*). HDAC11 was originally identified to be highly expressed in kidney, heart, brain, skeletal muscle, and testis (*17*). Advances highlight a role for HDAC11 in adipocyte gene expression control (Fig. 8). In adipocytes, HDAC11 was found to catalyze the removal of *N-*myristoyl groups from lysine residues on the cytoplasmic scaffolding protein Gravin-α (AKAP12) (*188*). AKAP12 lysine myristoylation resulted in the activation of protein kinase A signaling that increased thermogenic gene expression (*188*). Separately, in mice and ex vivo human adipose tissue models, the HDAC11 inhibitor FT895 was shown to promote the expression of *UCP1* via maintained AKAP12 lysine myristoylation (*189*). Together, findings suggest that HDAC11 inhibition in adipocytes could be used to manipulate thermogenesis (*188*, *189*). Specific HDAC11 isoform targeting may help to safely elicit greater therapeutic benefit than pan-HDAC strategies when seeking to manipulate HDAC11-dependent thermogenesis.

### Concluding remarks and future perspectives

Findings continue to uncover specific disease contexts where targeting HDACs holds therapeutic promise. HDAC inhibitors have historically targeted the catalytic activity of all 11 zinc-dependent HDACs, but this therapeutic approach has been met with limited success owing to limiting on-target toxicity, lack of clinically viable, isoform-specific HDAC inhibitors, and failure to elicit desired therapeutic response when used as single-agent therapeutics. It is now abundantly evident that successfully targeting the HDACs to elicit therapeutic benefit will require consideration of individual HDACs, subclasses of HDACs, and their interacting multiprotein complexes across a breadth of diseases. Recent studies have also suggested that HDACs may regulate gene expression by catalyzing acyl PTMs besides deacetylation, such as delactylation or decrotonylation, and future therapeutic strategies may consider targeting this acyl PTM-specific HDAC activity.

This review has spanned a breadth of tissue and disease contexts where the nonredundant zinc-dependent HDACs regulate gene expression through myriad mechanisms. While HDAC1 and HDAC2 remain the canonical HDACs of the HDAC superfamily, mechanisms of action beyond histone deacetylation have been reported. Emerging areas of focus include the nervous system, immune cells, and cancer. If appropriate therapeutic windows can be determined for inhibitors of these global chromatin modifiers, there are multiple disease contexts where HDAC1/HDAC2 inhibition could be impactful. In contrast, HDAC3-regulated gene expression programs impart potent regulation of key biological processes via a more restricted set of genes. Selective inhibition of HDAC3 in specific cancer types and combination therapy approaches hold therapeutic promise. Other emerging themes indicate relevance of HDAC3 in disorders of the nervous system, intestinal homeostasis, and immune cells. HDAC3-selective inhibitors would likely be well tolerated because of the comparatively smaller number of target genes coupled with unique mechanisms of action; HDAC3 is an up-and-coming target in disease therapy. Comparatively understudied compared to the other class I HDACs as of late, interplay between HDAC8 and HIF-1α has emerged as a theme across multiple tissue contexts. Among class II HDACs, phosphorylation has continued to prove itself as a dominant upstream regulator of the pleiotropic impacts of the class IIa HDACs across biological systems. The well-conserved biochemical link between signaling and class IIa HDACs may provide a promising opportunity for targeting class IIa HDAC-dependent pathologies, such as nervous system dysregulation, including sleep, addiction, and pain, cardiovascular disease, or cancer. Previously unidentified roles for HDAC6 in cancer suggest therapeutic relevance, particularly with the development of HDAC6 inhibitors with improved safety profiles, and HDAC10 has been implicated as a regulator of antitumor immunity. Last, previously unidentified roles for HDAC11 in adipocyte gene expression control have been uncovered.

Since 2020, findings continue to illustrate the breadth of mechanisms, physiological contexts, and disease states potently regulated by individual subfamilies and isoforms of the zinc-dependent HDAC1 to HDAC11. If findings are to be translated into effective disease therapy with limited on-target toxicity, attention must be paid to the roles of specific HDAC isoforms in particular tissue contexts; the devil is in the details. Excitingly, for certain tissue and disease contexts, these details are coming into focus, making now a promising time for the HDAC field.
